# Multilocus sequence typing (MLST) analysis reveals many novel genotypes and a high level of genetic diversity in *Candida tropicalis* isolates from Italy and Africa

**DOI:** 10.1111/myc.13483

**Published:** 2022-07-07

**Authors:** Aude Ngueguim Dougue, Mohammed A. El‐Kholy, Letterio Giuffrè, Grazia Galeano, Francesco D′Aleo, Cyrille Levis Kountchou, Claude Nangwat, Jean Paul Dzoyem, Domenico Giosa, Ida Pernice, Sherine M. Shawky, Thierry Kammalac Ngouana, Fabrice Fekam Boyom, Orazio Romeo

**Affiliations:** ^1^ Antimicrobial & Biocontrol Agents Unit (AmBcAU), Laboratory for Phytobiochemistry and Medicinal Plants Studies, Department of Biochemistry, Faculty of Science University of Yaoundé I Yaoundé Cameroon; ^2^ Department of Microbiology and Biotechnology, Division of Clinical and Biological Sciences, College of Pharmacy Arab Academy for Science, Technology and Maritime Transport (AASTMT) Alexandria Egypt; ^3^ Department of Chemical, Biological, Pharmaceutical and Environmental Sciences University of Messina Messina Italy; ^4^ Department of Veterinary Sciences University of Messina Messina Italy; ^5^ Laboratory of Clinical Microbiology Great Metropolitan Hospital of Reggio Calabria Reggio Calabria Italy; ^6^ Research Unit of Laboratory of Microbiology and Antimicrobial Substances (RUMAS), Department of Biochemistry, Faculty of Science University of Dschang Dschang Cameroon; ^7^ Institute of Medical Research and Medicinal Plant Studies Center for Medical Research in Health and Priority Pathologies Yaoundé Cameroon; ^8^ Department of Microbiology, Medical Research Institute University of Alexandria Alexandria Egypt; ^9^ Biomedical Research Unit Laboratoire Sion Yaoundé Cameroon

**Keywords:** *Candida tropicalis*, fluconazole resistance, genetic diversity, non‐*albicans Candida* species, candidiasis, multilocus sequence typing (MLST), population structure

## Abstract

**Background:**

*Candida tropicalis* is a human pathogenic yeast frequently isolated in Latin America and Asian‐Pacific regions, although recent studies showed that it is also becoming increasingly widespread throughout several African and south‐European countries. Nevertheless, relatively little is known about its global patterns of genetic variation as most of existing multilocus sequence typing (MLST) data come from Asia and there are no genotyped African isolates.

**Objectives:**

We report detailed genotyping data from a large set of *C. tropicalis* isolates recovered from different clinical sources in Italy, Egypt and Cameroon in order to expand the allele/genotype library of MLST database (https://pubmlst.org/ctropicalis), and to explore the genetic diversity in this species.

**Methods:**

A total of 103 *C. tropicalis* isolates were genotyped using the MLST scheme developed for this species. All isolates were also tested for in vitro susceptibility to various antifungals to assess whether certain genotypes were associated with drug‐resistance.

**Results and Conclusions:**

A total of 104 different alleles were detected across the MLST‐loci investigated. The allelic diversity found at these loci resulted in 51 unique MLST genotypes of which 36 (70.6%) were novel. Global optimal eBURST analysis identified 18 clonal complexes (CCs) and confirm the existence of a specific Italian‐cluster (CC36). Three CCs were also statistically associated with fluconazole resistance, which was elevated in Cameroon and Egypt.

Our data show high genetic diversity in our isolates suggesting that the global population structure of *C. tropicalis* is still poorly understood. Moreover, its clinical impact in Italy, Egypt and Cameroon appears to be relevant and should be carefully considered.

## 1 INTRODUCTION

Averting the threat of new infectious diseases has always been the main aim of several international health agencies^1^ but the recent coronavirus disease 2019 (COVID‐19) pandemic^2^ has undoubtedly raised awareness of the risk associated with microbial infections and emphasised the need to pay more attention to the identification, transmission and control of pathogenic microbes.^3^

However, in this global health emergency, another alarming scenario, involving microbes of the so‐called ‘hidden kingdom’, is emerging clearly from recent studies.^4^, ^5^ These are fungal infections that silently kill millions of people every year^6^ and cause huge economic losses for healthcare facilities.^7^ Undoubtedly, invasive candidiasis makes a substantial contribution to the total burden of fungal infections as demonstrated by over 750,000 cases reported every year worldwide.^4^ Of these, an estimated 400,000 cases are attributable to *Candida* bloodstream infection (BSI)^6^ which represents a serious growing concern given the high overall mortality rate observed (20–49%),^8^ that is likely to reach 70% at 30‐day in very old patients.^9^

*Candida* species are a heterogeneous group of phylogenetically diverse ascomycetous yeast, consisting of plant endophytes, insect symbionts and mammalian‐associated opportunistic pathogens which are capable of causing serious life‐threatening infections in humans.^10^ Currently, over 40 *Candida* species are known as aetiological agents of candidiasis^11^ and more than 95% of the infections are attributable to just five species (*Candida albicans*, *Candida glabrata*, *Candida tropicalis, Candida parapsilosis* and *Pichia kudriavzevii* [formerly *Candida krusei*])^12^ of which *C. albicans* remains the most important and most frequently recovered in both adult and paediatric patient populations.^13^ Nevertheless, non‐*albicans Candida* (NAC) species are increasingly isolated worldwide, and their incidence may even exceed that of *C. albicans* in some geographical regions^14^, ^15^, ^16^ and/or in certain clinical samples.^17^, ^18^ Among NAC species, *C. tropicalis* has emerged as important cause of BSIs, especially in aged and cancer patients^19^, ^20^, ^21^ and although previous studies have generally indicated that most of the infections occur in Latin America and Asian Pacific region,^14^, ^21^, ^22^ more recent epidemiological investigations revealed that *C. tropicalis* is also becoming locally prevalent in other geographical areas, especially in countries of the Mediterranean area of Europe and Africa.^15^, ^16^, ^23^, ^24^ Nevertheless, few studies regarding genetic diversity and potential association between different *C. tropicalis* genotypes and antifungal resistance, specimens, patient type or geographical locations have been performed so far, especially from African countries.^25^

Genetically, *C. tropicalis* is relatively similar to *C. albicans* ^26^ with which it shares also several phenotypic characteristics, including various virulence traits.^27^ The population genetic structure of these species, as evidenced by multilocus sequence typing (MLST) studies, also appears to be comparable,^28^ but unlike *C. albicans*, MLST data currently available for *C. tropicalis* are far less abundant and geographically biased.^25^ In fact, there are no African isolates deposited in the *C. tropicalis* MLST database (https://pubmlst.org/ctropicalis) and over 90% of the countries in the world are not represented.^25^ For these reasons, we decided to perform a retrospective study by genotyping a large set of *C. tropicalis* isolates recovered from different clinical sources in Italy, Egypt and Cameroon in order to expand the allele library of the central MLST database, and to explore the global patterns of genetic variation, evolution and molecular epidemiology in geographically diverse *C. tropicalis* populations.

## 2 MATERIALS AND METHODS

### 2.1 Yeast isolates, phenotypic and molecular identification

A total of 97 clinical *C. tropicalis* isolates from Italy (11), Egypt (61) and Cameroon (25) were retrospectively examined in this study (Table 1). All clinical isolates were initially phenotypically identified as part of the ordinary activity of the diagnostic microbiology laboratories of the respective hospitals/institutions involved in the study and stored at −80°C. Additional 6 environmental/clinical reference isolates from Westerdijk Fungal Biodiversity Institute (CBS‐KNAW biobank), recovered from countries not represented in the *C. tropicalis* MLST database, were also included (Table 1).

**TABLE 1:** *Candida tropicalis* isolates, allelic profiles and DSTs generated by MLST analysis

Isolate code	MLST loci						DST	CC
	*ICL1*	*MDR1*	*SAPT2*	*SAPT4*	*XYR1*	*ZWF1*		
CBS 13074, CBS 13076, CTEGY‐49	1	6	3	5	5	3	6	S
CTRC‐11, CTEGY‐2, CTEGY‐4, CTEGY‐5, CTEGY‐15, CTEGY‐16, CTEGY‐19, CTEGY‐23, CTEGY‐27, CTEGY‐33, CTEGY‐34, CTEGY‐37, CTEGY‐39, CTEGY‐45, CTEGY‐53, CTEGY‐54, CTEGY‐69, CTEGY‐72	1	4	22	23	36	9	139	4
CTEGY‐13	1	3	3	17	54	3	140	1
CTEGY‐50	1	3	3	17	57	3	168	1
CTEGY‐30	1	4	12	23	36	9	184	4
CTEGY‐35, CTEGY‐38, CTEGY‐44	1	7	1	6	52	4	237	2
CTEGY‐6, CTEGY‐9	1	22	12	17	60	22	331	5
CTCMR‐10	3	4	1	41	77	4	346	15
CTRC‐2, CTRC‐3	1	17	2	14	100	3	359	9
CTEGY‐1, CTEGY‐24, CTEGY‐28, CTEGY‐52, CTEGY‐56, CTEGY‐57, CTEGY‐62, CTEGY‐67, CTEGY‐68, CTEGY‐71	1	9	22	23	36	9	401	4
CTCMR‐14, CTCMR‐19	1	44	1	7	58	3	522	6
CTEGY‐17, CTEGY‐21	1	7	3	6	52	4	682	2
CTEGY‐31	1	42	1	23	54	1	689	62
CBS 2313	3	58	1	3	34	3	903^a^	S
CBS 2317	3	9	3	8	60	6	904^a^	106
CBS 13075, CBS 13077	10	19	60^a^	101^a^	159^a^	58^a^	905^a^	S
CTRC‐5, CTRC‐6	1	7	3	17	54	3	911	1
CTRC‐1	1	49	10	1	161^a^	1	915^a^	107
CTRC‐7	3	3	12	10	16	3	916^a^	108
CTRC‐4	47^a^	93	29	82	138	30	917^a^	36
CTRC‐8	1	1	2	23	54	1	918^a^	S
CTRC‐9	3	3	12	10	54	3	919^a^	108
CTRC‐10	15	91	29	102^a^	105	38	920^a^	109
CTEGY‐18	3	4	3	41	77	4	985	15
CTEGY‐14	3	22	68^a^	3	27	1	1162^a^	29
CTEGY‐20	1	169	2	10	68	1	1163^a^	S
CTEGY‐22	1	59	1	3	3	1	1164^a^	30
CTEGY‐7, CTEGY‐8, CTEGY‐25, CTEGY‐29, CTEGY‐40	1	42	1	7	54	1	1165^a^	62
CTEGY‐26	1	7	1	6	144	4	1166^a^	2
CTEGY‐32	1	7	3	7	76	6	1167^a^	7
CTEGY‐10, CTEGY‐36, CTEGY‐51	1	92	1	10	1	3	1168^a^	S
CTEGY‐41, CTEGY‐65	1	32	22	23	36	9	1169^a^	4
CTEGY‐42	10	195^a^	3	42	205^a^	44	1170^a^	S
CTEGY‐55	3	7	3	6	52	4	1171^a^	2
CTEGY‐61	5	53	1	34	69	9	1172^a^	S
CTEGY‐63	1	7	3	10	76	6	1173^a^	7
CTEGY‐73	1	196^a^	3	7	76	4	1174^a^	S
CTEGY‐74, CTCMR‐5	1	7	3	10	73	6	1175^a^	7
CTCMR‐2	1	7	3	7	73	6	1176^a^	7
CTCMR‐6, CTCMR‐7	1	76	1	10	73	40	1177^a^	S
CTCMR‐9, CTCMR‐12	1	3	3	11	175	6	1178^a^	S
CTCMR‐13, CTCMR‐15, CTCMR‐46, CTCMR‐50, CTCMR‐51, CTCMR‐58	1	39	3	7	80	17	1179^a^	64
CTCMR‐17	1	26	1	7	206^a^	3	1180^a^	S
CTCMR‐18	1	3	1	11	24	10	1181^a^	S
CTCMR‐20	1	44	1	7	58	6	1182^a^	6
CTCMR‐21	1	7	1	7	207^a^	1	1183^a^	S
CTCMR‐47	1	197^a^	1	10	48	1	1184^a^	S
CTCMR‐40	1	39	3	7	80	9	1185^a^	64
CTCMR‐3, CTCMR‐4	3	148	12	11	74	3	1186^a^	63
CTCMR‐11	3	148	12	11	175	6	1195^a^	S
CTCMR‐16	3	198^a^	12	11	74	3	1196^a^	63

Abbreviations: DST, Diploid sequence type; CC, Clonal Complex; S, Singleton.

^a^ New alleles/DSTs detected in this study; CTCMR delineates isolates from Cameroon, CTEGY from Egypt and CTRC from Italy.

Before molecular characterisation and MLST genotyping, the identity of all *C. tropicalis* isolates was phenotypically confirmed by subculture on CHROMagar *Candida* medium (CHROMagar, France). Definitive species identification was established by using a simple PCR‐based assay developed for rapid and easy identification of *C. tropicalis*.^29^ This method is based on the specie‐specific amplification of a 245 bp DNA fragment from the vacuolar membrane ATPase (VMA) intein gene using only a single pair of primers: VMA‐f: AATCCGAAGGCTTGATGG and VMA‐r: AATGCCAGCAGCAAAAGTAG.^29^

Genomic DNA was isolated according to Müller et al., 1998^30^ by using the high‐speed glass bead‐beating method combined with the conventional phenol‐chloroform‐isoamyl alcohol extraction and ethanol precipitation. In vitro amplifications were carried out in 50 μl reaction volumes using the DreamTaq Green PCR master mix (Thermo Fisher Scientific, Milan, Italy) supplemented with 0.5 μg of genomic DNA template and 0.5 μM of each VMA primer described above.^29^

PCRs were performed in a MyCycler thermal cycler (Bio‐Rad, Milan, Italy) using the following conditions: initial denaturation at 95°C for 5 min, followed by 30 cycles of denaturation at 94°C for 1 min, annealing at 53°C for 40 sec and extension at 72°C for 45 sec, and a final extension step of 7 min at 72°C. PCR products were then analysed by 2% w/v agarose gel electrophoresis to confirm the presence of the expected 245 bp amplicon.^29^

### 2.2 Multilocus sequence typing of *C. tropicalis* isolates

All 103 clinical and environmental *C. tropicalis* isolates were genotyped using the official MLST scheme developed for this species.^28^ MLST analysis was based on partial PCR amplification and DNA sequencing of the following six housekeeping genes *ICL1*, *MDR1*, *SAPT2*, *SAPT4*, *XYR1* and *ZWF1a* as previously described by Tavanti et al., 2005.^28^

For each isolate, six separate PCR amplifications were carried out in 50 μl using the Premix TaKaRa Taq version 2.0 DNA polymerase (TaKaRa, Italy), 100 ng of genomic DNA template and 1 μM (final concentration) of each primer.^28^ The MLST locus‐specific primers and cycling conditions used for PCR amplifications were the same as those previously reported by Tavanti et al., 2005.^28^ After PCR, each PCR product was first analysed by 1.5% w/v agarose gel electrophoresis to verify the presence/size of the expected amplicon and then purified by using the ExoSAP‐IT PCR Product Cleanup Reagent (Thermo Fisher Scientific, Milan, Italy). Purified PCR products were bi‐directionally sequenced at the Eurofins Genomics, Ebersberg, Germany (www.eurofinsgenomics.eu) using standard Sanger sequencing and the same MLST locus‐specific primers used for PCR.

Raw sequencing electropherograms were visually inspected using the FinchTV v1.4 software (GeoSpiza Inc., Seattle, WA) in order to detect base‐calling errors and potential heterozygous polymorphisms. DNA consensus sequences, including single‐nucleotide polymorphisms (SNPs), were first confirmed by matching both forward and reverse sequencing traces, and then edited as text‐based FASTA format according the single‐letter nucleotide code of the International Union of Pure and Applied Chemistry (IUPAC) nomenclature. The sequences from each MLST locus were compared with the respective reference sequences deposited into the central *C. tropicalis* MLST database (https://pubmlst.org/ctropicalis) for the assignment of allele numbers, which were used to define a diploid sequence type (DST), or MLST genotype, for each *C. tropicalis* isolate. Unassigned allele sequences were submitted to the MLST database (https://pubmlst.org/ctropicalis) and new allele numbers, including new DSTs, were provided by the curator: Prof. Hsiu‐Jung Lo.

### 2.3 Phylogenetic analysis and global population structure of *C. tropicalis*

Phylogenetic relationships among *C. tropicalis* isolates were inferred using the unweighted pair‐group method with arithmetic average (UPGMA) analysis of concatenated MLST sequences according to previous studies.^15^, ^31^ Additional 1269 validated isolates, available in the MLST database (https://pubmlst.org/ctropicalis; as of 26 May 2021), were included in the phylogenetic analysis. Moreover, a minimum spanning (MS) tree was generated from concatenated DSTs sequences using the GrapeTree software (https://achtman‐lab.github.io/GrapeTree).

To explore patterns of evolutionary descent among isolates, we divided the entire fungal population into groups of genetically closely‐related isolates termed clonal complexes (CCs). The CCs, including founder DSTs, were determined by global optimal e‐BURST (goeBURST) analysis using the PHYLOViZ 2.0 software.^32^ Isolates were considered as belonging to the same CC if sharing 5 out of 6 genes used for MLST.

### 2.4 Antifungal drug susceptibility testing of *C. tropicalis* isolates

The susceptibility of Egyptian and Italian *C*. *tropicalis* isolates to six antifungal drugs (amphotericin B, caspofungin, fluconazole, 5‐flucytosine, micafungin and voriconazole) was determined by using the VITEK 2 system (AST‐YS07 card) according to the manufacturer's instructions (bioMérieux, France). The susceptibility to antifungals of the Cameroonian isolates was instead performed using the Sensititre™ YeastOne™ system (Thermo Scientific) with nine drugs (anidulafungin, micafungin, caspofungin, amphotericin B, 5‐flucytosine, posaconazole, voriconazole, itraconazole, fluconazole) (Table S1). The results were interpreted using species‐specific clinical laboratory standards institute (CLSI) breakpoints (CBPs) as recommended by the M60‐ED2 document.^33^ However, where there were no CLSI CBPs (amphotericin B, Itraconazole, flucytosine and posaconazole),^33^ species‐specific epidemiological cut‐off values (ECVs) were used for distinguishing between wild‐type and non‐wild‐type isolates (those with acquired known resistance mechanisms).^34^, ^35^ Table S1 reports the CLSI CBPs and ECVs used in this study.

### 2.5 Statistical analysis

All statistical analyses were performed using the R package stats (version 4.0.2) downloaded from CRAN (https://cran.r‐project.org). Categorical variables were expressed as absolute frequencies and/or percentages (*n*; %). The statistical significance of an association between two groups (i.e., clonal clusters or DSTs and antifungal susceptibility data) was estimated by using the chi‐square or Fisher's exact test. If *p*‐values were ≤.05, differences between groups were considered statistically significant.

The statistical analysis included the minimal inhibitory concentration (MIC) values obtained for all *C. tropicalis* isolates tested in this study and antifungal susceptibility data of 482 isolates for which at least a MIC value was submitted in the central *C. tropicalis* MLST database (https://pubmlst.org/ctropicalis).

## 3 RESULTS

### 3.1 Patient data, fungal isolates, phenotypic and molecular identification

In this study, most of the *C. tropicalis* clinical isolates were recovered from urine samples (*n*° 52), followed by blood (*n*° 14), stool (*n*° 10), sputum (*n*° 8), vaginal (*n*° 7), oral (*n*° 2) and other specimens, including medical devices (Table S1). Clinical metadata from the 25 Cameroonian patients show that most of them were HIV‐positive (19/25; 76%) whereas 6 (24%) were type‐2 diabetic patients. Unfortunately, no data about the underlying health conditions of the Egyptian patients were available, while for the Italian ones 4 were reported to be suffering from cancer (Table S1).

The patient ages ranged from 2 to 90 years (mean 57.9 ± SD 15.6), and only less than one third of them (29/97; 29.9%) were ≥65 years old. The ratio of male to female was 1.425:1 (57 males and 40 females) (Table S1).

All fungal isolates included in this study produced blue colonies when sub‐cultured on CHROMagar *Candida* medium at 37°C for 24/48 h. PCR amplification of the VMA‐intein gene showed the presence of the expected 245 bp‐amplicon in all isolates by confirming their identity as *C. tropicalis*.

### 3.2 MLST genotyping and genetic diversity of *C. tropicalis* isolates

Sequence analysis of 370–525 bp DNA fragments, obtained from the six gene targets included in the MLST typing scheme for *C. tropicalis*, yielded a total of 2677 aligned nucleotides for each isolate.

A total of 104 different alleles were detected across the six MLST loci investigated and the *XYR1* gene was the most polymorphic (31 alleles), while the *ICL1* gene was the least informative locus (6 alleles) (Table 2). Over 14% (15/104) of the observed alleles were new and were identified in DNA fragments obtained from all MLST loci: *ICL1* (allele 47), *MDR1* (alleles 195, 196, 197 and 198), *SAPT2* (alleles 60 and 68), *SAPT4* (alleles 101 and 102), *XYR1* (alleles 159, 161, 205, 206 and 207) and *ZWF1a* (allele 58) (Table 1). Overall, 173 polymorphic sites were identified in our isolates, corresponding to a ratio of 6.5% (173/2677 nucleotides) when including all housekeeping genes (Table 2). The number of variable nucleotide sites at each locus ranged from 15 (*ICL1* locus) to 50 (*SAPT4* locus) (Table 2) indicating a considerable amount of genetic diversity for each of the loci examined. The allelic diversity found at these MLST loci resulted in a total of 51 unique DSTs of which 36 (~70.6%; 36/51) were novel to the *C. tropicalis* MLST database.

**TABLE 2:** Characteristics of the six MLST loci examined in this study

MLST Locus	Sequenced fragment size (bp)	*N*° of alleles	*N*° of variable sites	Nucleotide position
*ICL1*	447	6	15	43,58,61,76,79,124,169,217,220,256,271,277,391,421,436
*MDR1*	425	28	24	4,13,44,55,70,97,157,178,223,242,247,250,262,277,310,319,337,340,343,367,371,394,403,421
*SAPT2*	525	9	41	5,32,35,38,50,78,102,110,125,131,138,140,143,172,206,209,236,238,239,248,254,256,267,269,288,315,316,323,338,356,368,377,389,398,418,455,461,479,515,519,520
*SAPT4*	390	17	50	4,13,26,37,43,45,61,64,73,97,103,104,106,109,113,115,130,133,163,169,170,172,193,195,196,213,220,226,229,239,250,259,268,271,277,292,300,307,313,322,328,347,370,371,376,378,379,380,382,388
*XYR1*	370	31	26	11,14,44,59,65,83,92,101,104,125,128,129,131,134,137,149,158,180,188,215,218,242,287,303,341,344
*ZWF1a*	520	13	17	28,43,52,61,64,73,97,113,157,163,229,238,304,355,376,505,508
**Total**	**2677**	**104**	**173**	‐

The bold values simply indicate the total of each column.

Globally, the most frequent MLST genotype was DST139 (18/103; 17.5%), followed by DST401 (10/103; 9.7%), DST1179 (6/103; 5.8%) and DST1165 (5/103; 4.8%) (Table 1). Fourteen different genotypes were shared by two or three isolates while the remaining 33 DSTs were unique to single isolates (singletons) and showed a relative frequency of less than 1% each (Table 1).

Interestingly, except DST1179, which included fungal isolates geographically restricted to Cameroon, the other three most frequent genotypes (DSTs 139, 401 and 1165) comprised more than half of the *C. tropicalis* isolates recovered in Egypt (32/61 isolates; ~52.5%) (Table 1).

Overall, we observed a differential distribution of DSTs between the three countries (Egypt, Cameroon and Italy) with 15 DSTs belonging only to *C. tropicalis* isolates from Cameroon, 22 DSTs to the Egyptian ones and 8 DSTs exclusively associated with Italian isolates (Table 1). Only three genotypes (DSTs 6, 139 and 1175) were shared by temporally and geographically diverse isolates, and therefore, although epidemiologically unrelated, they were genetically indistinguishable by the MLST scheme employed in this study.

**Figure 1:**
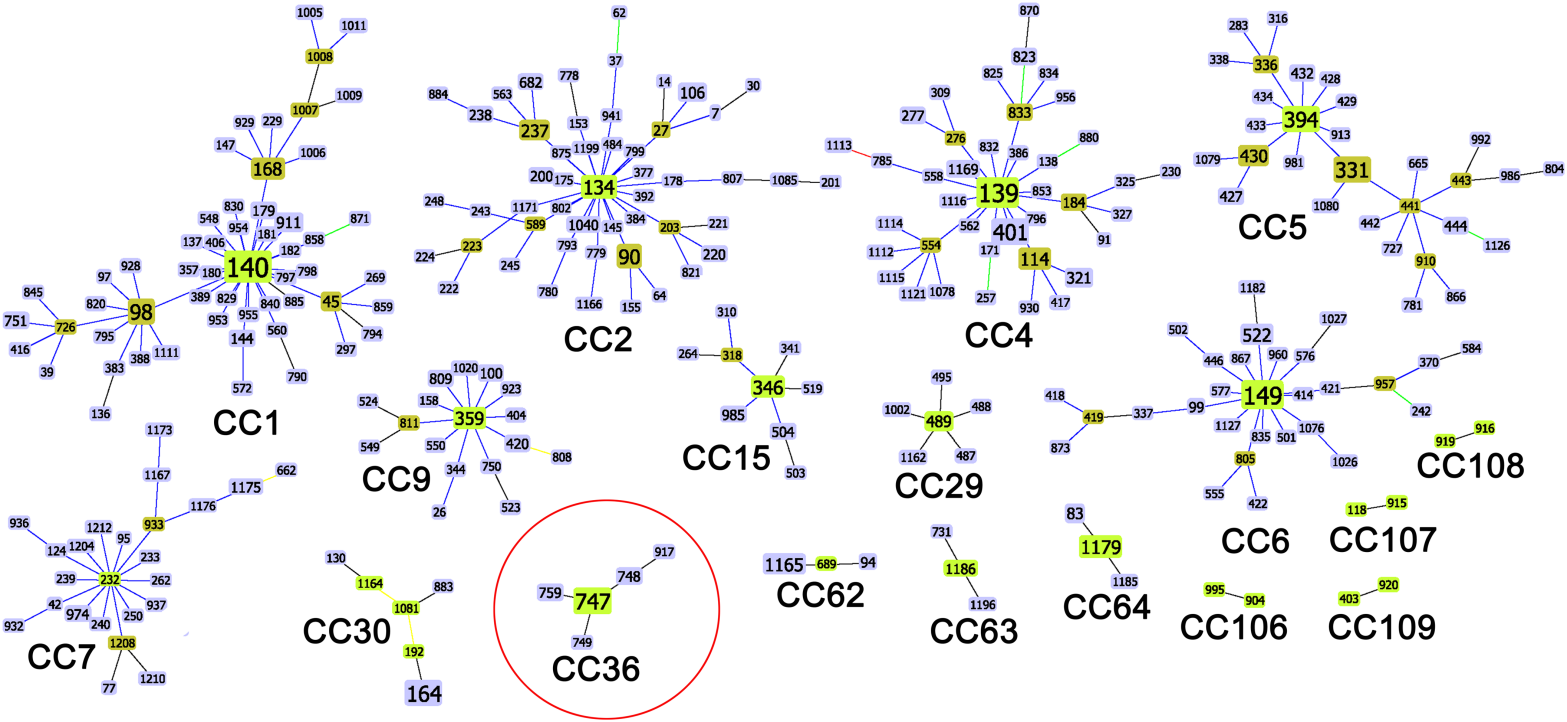
Details of the 18 goeBURST clonal complexes detected in this study. For each cluster, the total number of DSTs is shown. Putative founder genotypes (green) are positioned centrally in each cluster, and subgroup founders are shown in olive green. The size of each rectangle, containing the DST code, reflects the number of isolates with that MLST genotype in the whole dataset employed for the analysis. The lengths of lines are not significant. The clonal complex circled in red (CC36) represents the exclusive genetic cluster found in southern Italy

The MLST data were further analysed for inferring genetic relationships between our isolates and those deposited in the official *C*. *tropicalis* MLST database (1269 isolates; as of 26 May 2021). The MLST dataset contained 986 confirmed DSTs that were clustered into 109 CCs by their similarity to a central founding genotype and 326 singletons. Eighteen clusters (CCs 1, 2, 4, 5, 6, 7, 9, 15, 29, 30, 36, 62, 63, 64, 106, 107, 108 and 109) (Figure 1) contained most of the isolates (80/103; 77.7%) examined in this study, while 23 isolates (22,3%) were singletons (Table 1).

The whole dataset with all the 18 CCs found in this study, including all the isolates, countries, continents and DSTs that characterise each CC, is shown in the Table S2 and is also available online on figshare through the following link https://figshare.com/s/ee7b6e9bfca3c56543b6.

The two most frequent genotypes from Egypt (DSTs 139 and 401) were grouped in the CC4 (Figure 1) which also contained 40 DSTs and 82 isolates with a worldwide distribution, mainly from Asia. Other Egyptian isolates (DTSs 237, 682, 1166 and 1171) were found within another heterogeneous clonal complex (CC2) (Figure 1), together with a number of DSTs and isolates of different geographic origin (Asia, Europe, North and South America). On the contrary, CC62 was geographically more homogeneous as it contained a total of eight isolates of which six from Egypt (DST 1165 and 689) and two from the UK (DST94) (Table 1; Figure 1).

The most populated clonal complex (CC1) comprised 99 isolates and 55 DSTs, including three MLST genotypes (DSTs 168, 140 and 911) described in this study (Figure 1). The DST140, recovered from the Egyptian isolate CTEGY‐13, was predicted as the putative founding genotype of this clonal complex. Among the genotypes identified in Cameroon, the most frequent type (DST1179) was the putative founding genotype of CC64 complex which also includes the DST1185 genotype from the same country, and one genotype (DST83) previously identified in the UK (Figure 1). Moreover, an additional Cameroonian genotype (DST1186) was found to be the founder genotype of CC63 which also contains the DST731 isolated in China (Figure 1).

Among the genotypes obtained from reference CBS isolates, only the DST904, obtained from the Russian CBS 2317 isolate, was included in a clonal complex (CC106) together with one other Asian genotype, the chinese DST995 (Figure 1).

**Figure 2:**
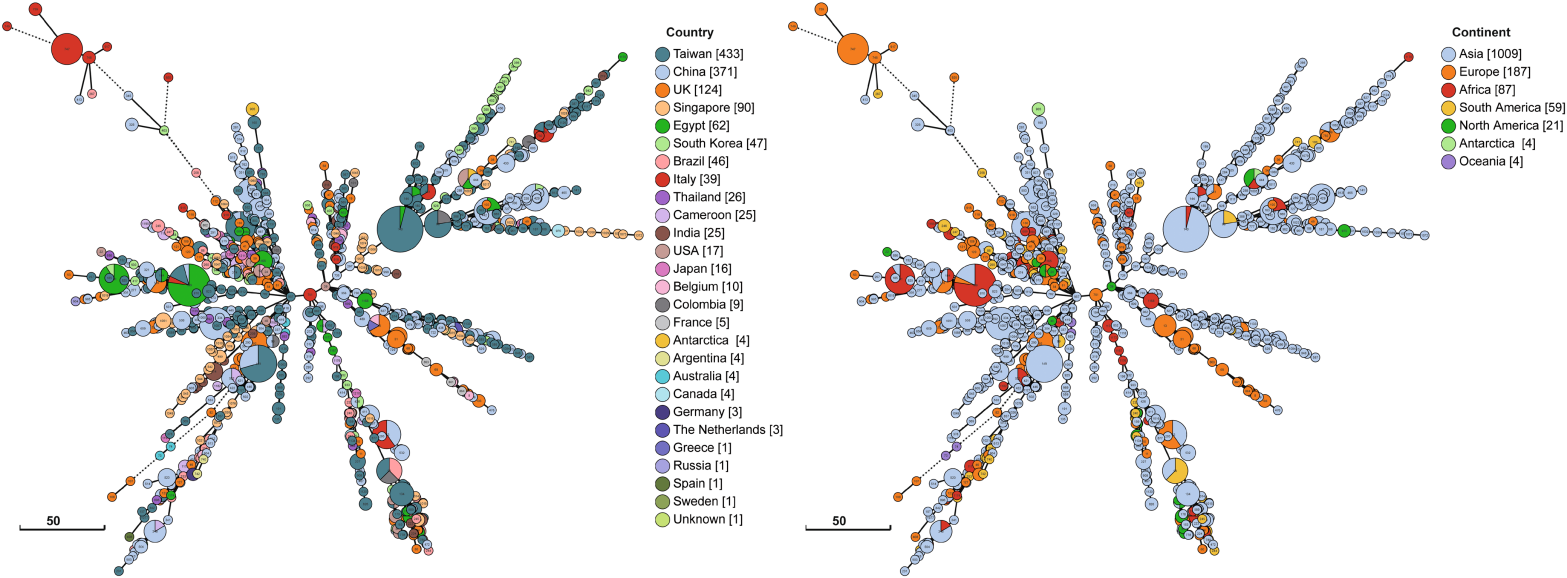
Minimum spanning tree showing the population structure of *Candida tropicalis* generated using the DSTs obtained in this study, including those of 1269 isolates deposited in the central MLST database. Each coloured circle represents a unique DST with the genotype code inside. The size of the circles reflects the number of isolates with that genotype, which is also shown in square brackets in the legends. The global distribution of the DSTs is displayed by country and by continent

The CC108 contained only genotypes recovered in Italy (DSTs 916 and 919), while the CC109 included the Italian DST920 and one isolate with DST403 from South Korea. Interestingly, the new Italian DST917 genotype reported in this study, clustered with other well‐known genotypes (DSTs 747, 748, 749 and 759) within the CC36 complex (Figure 1) which contains only fungal isolates from Italy. Phylogenetic analysis, based on UPGMA algorithm, confirmed that the DST917 belongs to the Italian lineage which was first detected in 2018^15^ and since then reported only from patients admitted to hospitals in Southern Italy. The minimum spanning tree, based on the analysis of MLST concatenated sequences, showed that this lineage is the most divergent and very distantly related to the rest of the isolates of the global population (Figure 2). However, all other *C. tropicalis* isolates from this study were scattered across the MS tree without any apparent geographical grouping (Figure 2).

### 3.3 Antifungal drug‐resistance and its potential association with MLST clonal complexes

The antifungal susceptibility pattern of each isolate is shown in the Table S1. Overall, we observed different levels of resistance to antifungal agents for which CBPs were defined with a clear variation in prevalence by geographic area (Table 3). In particular, among 97 *C. tropicalis* isolates tested, the highest level of resistance was observed for fluconazole (38/97 isolates; 39.2%) followed by voriconazole, caspofungin and micafungin (Table 3). For anidulafungin, although no data were available for Italian and Egyptian isolates, no resistance was observed in isolates from Cameroon. However, two Cameroonian isolates showed MIC values in the intermediate range (Table 3; Table S1).

**TABLE 3:** Antifungal susceptibility data obtained by testing various antifungal agents for which CLSI CBPs are currently available

Antifungal agent	N° of susceptible (S) and non‐susceptible (R, I and SDD) *C. tropicalis* isolates detected per country												Total isolates (97)^a^				% total R
	Italy (11)^a^				Egypt (61)^a^				Cameroon (25)^a^								
	R	I	SDD	S	R	I	SDD	S	R	I	SDD	S	R	I	SDD	S	
Fluconazole	0	0	0	11	24	0	3	34	14	0	2	9	38	0	5	54	~39.2
Voriconazole	0	0	0	11	6	20	0	35	6	13	0	6	12	33	0	52	~12.4
Caspofungin	0	0	0	11	1	0	0	60	2	3	0	20	3	3	0	91	~3.1
Micafungin	0	0	0	11	0	0	0	61	1	2	0	22	1	2	0	94	~1
Anidulafungin	ND	ND	ND	ND	ND	ND	ND	ND	0	2	0	23	‐‐	‐‐	‐‐	‐‐	‐‐

Abbreviations: I, intermediate; ND, Not Detected; R, resistant; S, susceptible; SDD, susceptible dose‐dependent.

^a^ The numbers in parentheses indicate the total number of isolates tested.

Cameroon was, in general, the country with the highest prevalence of non‐susceptible isolates, particularly against fluconazole and voriconazole whose resistance rates were remarkably high (56% and 24%, respectively) (Table 3; Table S1). Furthermore, for other azole drugs without defined CBPs (itraconazole and posaconazole), we found a high rate of MIC values exceeding their respective ECVs, suggesting that most of Cameroonian isolates may have acquired resistance to these drugs as well. Based on MIC data obtained and current ECVs, for posaconazole we classified 84% of Cameroonian isolates (21/25) as ‘non‐Wild Type’ (NWT) followed by itraconazole (11/25; 44% NWT) and 5‐fluorocytosine (1/25; 4% NWT) (Table S1).

A high detection rate of azole non‐susceptible isolates was also found in Egypt, where fluconazole resistance reaches over 39% of the tested isolates (24/61) (Table 3). Unfortunately, for Italian and Egyptian isolates, no data were available for antifungal drugs with published ECVs, except for amphotericin B where all *C. tropicalis* isolates, including those from Cameroon, were classified as ‘Wild Type’ (WT) (Table S1).

For 5‐fluorocytosine, the lower limit of the twofold dilution series in the VITEK 2 AST‐YS07 card stops at ≤1 μg/ml^36^ (Table S1), and therefore, for Egyptian and Italian isolates, it was not possible to classify them using the ECV available for this drug (0.5 μg/ml).^35^

Finally, no antifungal resistance or other non‐susceptible pattern was observed in isolates of Italian origin which is in agreement with the results of previous studies on similar genotypes^15^ and other reports.^37^, ^38^

In this study, potential association between MLST genotypes or CCs and non‐susceptibility to antifungal drugs was also evaluated using 482 out of 1269 isolates for which at least a MIC value was recorded in the *C. tropicalis* MLST database. Our statistical analysis showed no significant association (*p* > .05) between any CC and non‐susceptibility to antifungal agents such as caspofungin, amphotericin B itraconazole and posaconazole for which CBPs and/or ECVs were established.^33^, ^34^, ^35^ For anidulafungin and micafungin, we observed only one CC (CC7) and two CCs (CC7 and CC64), respectively, potentially associated with resistance to these drugs (*p* < .05). However, each of these CCs contained only one single resistant isolate, one from Canada (SP4501 isolate; DST974) in the CC7, resistant to both echinocandins, and one from Cameroon (CTCMR‐50 isolate; DST1179) in the CC64 that was found to be resistant to micafungin in this study (Table S1).

Regarding voriconazole, we did not observe any significant association between the established CCs and this drug (*p* > .05), while we found three clonal complexes statistically associated with resistance to fluconazole (CC1, *p* = .0000006; CC6, *p* = .0006; CC63, *p* = .026). These CCs contained a total of 40 (CC1), 16 (CC6) and 3 (CC63) fluconazole‐resistant isolates and most were from Asian origin. In fact, among all 59 fluconazole‐resistant isolates populating these CCs, 53 (~90%) were originally recovered in Taiwan and only 6 were from this study: the Egyptian CTEGY‐13 isolate (DST 140) belonging to CC1, two Cameroonian isolates CTCMR‐20 (DST 1182) and CTCMR‐14 (DST 522) found in CC6, and three additional Cameroonian isolates CTCMR‐3 and CTCMR‐4 (DST 1186) and CTCMR‐16 (DST1196) in CC63. However, contrary to what was observed for CCs, except for amphotericin B, we found a significant statistical association between genotypes and non‐susceptibility when global DSTs and MIC values were evaluated (Table S3). Notably, among 36 unique non‐susceptible DSTs, 6 new genotypes were from this study and were not sensitive (or not‐wild‐type) to one (DSTs 1175 and 1177) or more (DSTs 1176, 1179, 1184 and 1186) antimycotics. Interestingly, the Cameroonian DST 1179 and DST 1186 were associated with non‐susceptibility to at least two main classes of antifungal agents (azoles and echinocandins).

## 4 DISCUSSION

Today, the global emergence of invasive *Candida* infections is a serious and alarming problem that can no longer be ignored as pathogenic *Candida* species affect over 750,000 people every year worldwide.^4^

Recently, the SENTRY antifungal surveillance program published important epidemiological data concerning 20,788 clinical isolates of different *Candida* spp. collected during 20 years of surveillance from 135 medical centres in 39 countries around the world.^39^ Based on the SENTRY results, *C. albicans* still remains the most common cause of human candidiasis although its frequency decreased steadily from 57.4% in 1997–2001 to 46.4% in 2015–2016.^39^ In parallel, a worrying increase in the isolation of NAC species has been observed in many geographical regions^39^, ^40^, ^41^ and *C. tropicalis* appears to be the most frequently isolated species in Latin America and Asian Pacific region.^39^, ^41^, ^42^, ^43^ However, several studies documented the emergence of azole non‐susceptible *C. tropicalis* isolates in many other geographical areas such as Spain,^19^ Italy,^44^, ^45^ Turkey,^23^ Cameroon,^46^ Tanzania,^47^ Egypt,^48^ Tunisia,^24^, ^49^ Lebanon^50^ and Algeria,^16^ supporting several recent Asian reports that raise awareness of the growing problem of fluconazole resistance detected in this species.^31^, ^43^, ^51^, ^52^ Similarly, in this study, we confirm the occurrence of a high level of fluconazole resistance also in Egyptian and Cameroonian isolates, a phenomenon that is in line with the global increasing trend in resistance seen over the last years.^39^ This high level of resistance could be linked to the widespread use of this drug in these countries, especially in Cameroon where fluconazole is the only antifungal agent available.^53^

Interestingly, recent studies in China^31^, ^54^, ^55^ showed a putative association between some MLST clonal complexes and azole non‐susceptible *C. tropicalis* isolates. This correlation was also detected by microsatellite genotyping studies^42^ and was previously described also for other antifungals in France, where a flucytosine‐resistant clone was found to be widespread among hospitalised patients.^56^ Unique MLST genetic clusters, containing fluconazole non‐susceptible (FNS) *C. tropicalis* isolates, have been found in Singapore, India and China and have never been reported outside Asia^31^ although, in our study, we identified a specific MLST clonal complex (CC63) particularly enriched with fluconazole‐resistant isolates from Cameroon. Moreover, the large CC1 cluster was also statistically highly associated with fluconazole resistance and our data agree with previous findings reporting a close correlation between various CC1‐genotypes (DSTs 98, 137, 140 and 144) and drug‐resistant *C. tropicalis* clones in Taiwan.^57^, ^58^ Interestingly, goeBURST analysis also confirmed the presence of a well‐definite genetic cluster (CC36; Figure 1), containing only drug‐susceptible isolates recovered in southern Italy^15^ (Figure 1). However, in our opinion, all these MLST‐based association studies should be considered with caution given the presence of possible information/selection biases in the official *C. tropicalis* MLST database.^25^ In fact, current epidemiological data in the database are geographically highly skewed as over 78% of the isolates come from only one continent (Asia) followed by Europe (15.2%), South America (4.4%), North America (1.6%) and Oceania (0.3%) (http://pubmlst.org/ctropicalis; last accessed 04 February 2022). Unfortunately, there are currently no genetic data available from the entire African continent and therefore our study represents the first effort to expand geographic sampling and strain representation in the MLST database. Moreover, in addition to these data bias issues, which have a significant impact on the real amount of genetic diversity in the global *C. tropicalis* population,^25^ it should also be stressed that there is a remarkable lack of essential metadata, especially those from antifungal susceptibility testing. As per February 2022, a MIC value for fluconazole was present for only 39% (529/1351) of the isolates deposited, making MLST data underpowered to detect significant associations.^25^, ^59^ In this regard, the MLST genotypes (DSTs 225, 376, 506 and 546) of the large Chinese FNS clonal complex (CC2), recently described by Wang et al., 2020^31^ in Wuhan, were grouped by our goeBURST analysis with six fluconazole‐susceptible Italian isolates indicating that all the genetic diversity (not only genotypes with MIC data)^31^ should be considered to assess potential relationships between MLST genotypes and other variables, for example geographic locations. Furthermore, based on our data, the use of clonal complexes in association analyses may not be exhaustive as the results obtained by analysing global MIC values and MLST genotypes showed a positive correlation of some DSTs with non‐susceptibility to caspofungin (DST 1176 and 1186) or posaconazole (DST 1179), a feature that was not detected by statistical analysis employing CCs.

The global genetic structure of the *C. tropicalis* population has not yet been fully elucidated as its complexity grows rapidly as the number of different types of isolates (clinical or environmental) increases.^15^, ^37^, ^60^ Our MLST data confirm this trend, as 14% of MLST alleles and over 70% of DSTs detected here were novel (Table 1). A combination of four of the new alleles (alleles: 60 *SAPT2*; 101 *SAPT4*; 159 *XYR1* and 58 *ZWF1a*) was found in two reference CBS isolates (CBS 13075, CBS 13077; DST905), recovered from seawater in Antarctica, suggesting that genetic diversity, as well as patterns of variation, in environmental isolates are yet to be fully explored.^25^, ^60^ This highlights that future studies should be focused also on understanding the natural origin of *C. tropicalis* given the close genetic relationship found between clinical and animal/environmental isolates.^52^

The clinical and economic impact of candidiasis in Italy,^61^ Egypt^62^ and Cameroon^53^ is enormous and *C. tropicalis* could, in the future, become one of the most important NAC species in these countries. This assumption is seemingly well supported by recent epidemiological data from Algeria^16^ and several other Mediterranean countries^19^, ^23^, ^24^, ^44^, ^45^ which call particular attention, especially where fluconazole is the first‐line antifungal drug in prophylaxis and treatment of candidiasis.

In conclusion, we also believe that the amount of genetic information in the MLST database can be fully exploited only if other isolates of different origin (e.g., geographic, clinical and environmental) and high‐quality metadata are also collected and submitted. For example, MIC data for antifungals should be mandatorily submitted, together with other clinical and/or phenotypic information. This integrative approach, coupled with powerful bioinformatics tools, will pave new ways for fungal genetics by moving it from population studies to better management of invasive fungal infections and antifungal resistance in hospitals worldwide.

## AUTHOR CONTRIBUTIONS

**Aude Ngueguim Dougue:** Investigation (lead); Data curation (lead); Formal analysis (lead); Methodology (supporting). **Mohammed A. El‐Kholy:** Investigation (lead); Data curation (lead); Formal analysis (supporting). **Letterio Giuffrè:** Formal analysis (lead); Methodology (lead); Software (lead). **Grazia Galeano:** Formal analysis (equal); Methodology (supporting); Validation (lead)**. Francesco D'Aleo:** Investigation (lead); Data curation (supporting). **Cyrille Levis Kountchou:** Investigation (equal); Data curation (equal)**. Claude Nangwat:** Investigation (equal); Data curation (equal). **Jean Paul Dzoyem:** Conceptualization (lead); Supervision (lead). **Domenico Giosa:** Software (lead); Validation (supporting); Visualisation (lead); **Ida Pernice:** Resources (lead); Validation (supporting); Writing–original draft (supporting). **Sherine M. Shawky:** Conceptualization (supporting); Supervision (equal); Resources (supporting). **Thierry Kammalac Ngouana:** Conceptualization (lead); Supervision (lead); Writing–original draft preparation (supporting). **Fabrice Boyom Fekam:** Conceptualization (lead); Supervision (lead). **Orazio Romeo:** Conceptualization (lead); Supervision (lead); Funding acquisition (lead); Project administration (lead); Writing–original draft (lead); Writing–review and editing (lead).

## CONFLICT OF INTEREST

The authors declare that there are no conflicts of interest.

## Supporting information


Table S1

Table S2

Table S3

## Data Availability

All data generated in this study have been provided within the article or in supplementary data files, and are also publicly available and accessible online at C. tropicalis MLST database (https://pubmlst.org/ctropicalis). The datasets reported in the supplementary tables (Tables S1‐S3) are also available online on figshare and can be examined and/or downloaded using the following links: Table S1: https://figshare.com/s/fc548736d9efabb28bee (doi: 10.6084/m9.figshare.19137626) Table S2: https://figshare.com/s/ee7b6e9bfca3c56543b6 (doi: 10.6084/m9.figshare.19137602) Table S3: https://figshare.com/s/01ba53a6810f1a6c860d (doi: 10.6084/m9.figshare.19928477)
