# Supplementary material for: Multilocus sequence typing (MLST) analysis reveals many novel genotypes and a high level of genetic diversity in Candida tropicalis isolates from Italy and Africa
Source: Mycoses. 2022 Jul 7;65(11):989–1000. doi: 10.1111/myc.13483 (PMC9796097; doi:10.1111/myc.13483)
Supplement: Supplementary file 1 — Table S1 [file MYC-65-989-s002.docx]

| **Supplementary Table S1. Origin of *C. tropicalis* isolates, clinical metadata and MLST genotypes obtained in this study.** | | | | | | | | | |
| --- | --- | --- | --- | --- | --- | --- | --- | --- | --- |
| **Isolate code** | **MLST allelic profile** | **DSTs** | **CCs** | **Isolation year** | **Sample** | **Country** | **Patient's underlying disease** | **Sex** | **age (years)** |
|  | **(*ICL1-MDR1-SAPT2-SAPT4-XYR1-ZWF1a*)** |  |  |  |  |  |  |  |  |
| CTRC-1 | 1-49-10-1-161-1 | 915 | 107 | 2019 | Urine | Italy | N/A | F | 74 |
| CTRC-2 | 1-17-2-14-100-3 | 359 | 9 | 2018 | Blood | Italy | N/A | M | 62 |
| CTRC-3 | 1-17-2-14-100-3 | 359 | 9 | 2018 | Urine | Italy | N/A | M | 78 |
| CTRC-4 | 47-93-29-82-138-30 | 917 | 36 | 2018 | Bronchoalveolar lavage | Italy | Lung cancer | M | 82 |
| CTRC-5 | 1-7-3-17-54-3 | 911 | 1 | 2018 | Blood | Italy | Leukemia | M | 59 |
| CTRC-6 | 1-7-3-17-54-3 | 911 | 1 | 2019 | Blood | Italy | Leukemia | M | 64 |
| CTRC-7 | 3-3-12-10-16-3 | 916 | 108 | 2019 | Blood | Italy | Lymphoma | M | 76 |
| CTRC-8 | 1-1-2-23-54-1 | 918 | S | 2019 | Blood | Italy | N/A | F | 50 |
| CTRC-9 | 3-3-12-10-54-3 | 919 | 108 | 2019 | Urine | Italy | N/A | M | 68 |
| CTRC-10 | 15-91-29-102-105-38 | 920 | 109 | 2018 | Blood | Italy | N/A | M | 64 |
| CTRC-11 | 1-4-22-23-36-9 | 139 | 4 | 2018 | Blood | Italy | N/A | M | 71 |
| CTEGY-1 | 1-9-22-23-36-9 | 401 | 4 | 2014 | Urine | Egypt | N/A | M | 75 |
| CTEGY-2 | 1-4-22-23-36-9 | 139 | 4 | 2014 | Urine | Egypt | N/A | F | 63 |
| CTEGY-4 | 1-4-22-23-36-9 | 139 | 4 | 2014 | Urine | Egypt | N/A | F | 54 |
| CTEGY-5 | 1-4-22-23-36-9 | 139 | 4 | 2014 | Urine | Egypt | N/A | F | 85 |
| CTEGY-6 | 1-22-12-17-60-22 | 331 | 5 | 2014 | Urine | Egypt | N/A | M | 59 |
| CTEGY-7 | 1-42-1-7-54-1 | 1165 | 62 | 2014 | Urine | Egypt | N/A | M | 61 |
| CTEGY-8 | 1-42-1-7-54-1 | 1165 | 62 | 2014 | Urine | Egypt | N/A | M | 28 |
| CTEGY-9 | 1-22-12-17-60-22 | 331 | 5 | 2014 | Urine | Egypt | N/A | F | 65 |
| CTEGY-10 | 1-92-1-10-1-3 | 1168 | S | 2014 | Blood | Egypt | N/A | F | 2 |
| CTEGY-13 | 1-3-3-17-54-3 | 140 | 1 | 2014 | Sputum | Egypt | N/A | F | 42 |
| CTEGY-14 | 3-22-68-3-27-1 | 1162 | 29 | 2014 | Sputum | Egypt | N/A | F | 63 |
| CTEGY-15 | 1-4-22-23-36-9 | 139 | 4 | 2014 | Urine | Egypt | N/A | M | 59 |
| CTEGY-16 | 1-4-22-23-36-9 | 139 | 4 | 2014 | Foley's urinary catheter | Egypt | N/A | M | 81 |
| CTEGY-17 | 1-7-3-6-52-4 | 682 | 2 | 2014 | Blood | Egypt | N/A | M | 64 |
| CTEGY-18 | 3-4-3-41-77-4 | 985 | 15 | 2014 | Sputum | Egypt | N/A | M | 76 |
| CTEGY-19 | 1-4-22-23-36-9 | 139 | 4 | 2014 | Urine | Egypt | N/A | F | 73 |
| CTEGY-20 | 1-169-2-10-68-1 | 1163 | S | 2014 | Urine | Egypt | N/A | M | 88 |
| CTEGY-21 | 1-7-3-6-52-4 | 682 | 2 | 2014 | Urine | Egypt | N/A | M | 77 |
| CTEGY-22 | 1-59-1-3-3-1 | 1164 | 30 | 2014 | Urine | Egypt | N/A | M | 64 |
| CTEGY-23 | 1-4-22-23-36-9 | 139 | 4 | 2014 | Urine | Egypt | N/A | M | 65 |
| CTEGY-24 | 1-9-22-23-36-9 | 401 | 4 | 2014 | Urine | Egypt | N/A | F | 73 |
| CTEGY-25 | 1-42-1-7-54-1 | 1165 | 62 | 2014 | Blood | Egypt | N/A | M | 69 |
| CTEGY-26 | 1-7-1-6-144-4 | 1166 | 2 | 2014 | Urine | Egypt | N/A | M | 2 |
| CTEGY-27 | 1-4-22-23-36-9 | 139 | 4 | 2014 | Urine | Egypt | N/A | F | 57 |
| CTEGY-28 | 1-9-22-23-36-9 | 401 | 4 | 2014 | Sputum | Egypt | N/A | F | 67 |
| CTEGY-29 | 1-42-1-7-54-1 | 1165 | 62 | 2015 | Urine | Egypt | N/A | M | 54 |
| CTEGY-30 | 1-4-12-23-36-9 | 184 | 4 | 2015 | ETT (Endotracheal Tube) | Egypt | N/A | F | 77 |
| CTEGY-31 | 1-42-1-23-54-1 | 689 | 62 | 2015 | Urine | Egypt | N/A | M | 47 |
| CTEGY-32 | 1-7-3-7-76-6 | 1167 | 7 | 2015 | Blood | Egypt | N/A | M | 56 |
| CTEGY-33 | 1-4-22-23-36-9 | 139 | 4 | 2015 | Sputum | Egypt | N/A | M | 58 |
| CTEGY-34 | 1-4-22-23-36-9 | 139 | 4 | 2015 | Urine | Egypt | N/A | F | 81 |
| CTEGY-35 | 1-7-1-6-52-4 | 237 | 2 | 2015 | Urine | Egypt | N/A | F | 60 |
| CTEGY-36 | 1-92-1-10-1-3 | 1168 | S | 2015 | Blood | Egypt | N/A | F | 55 |
| CTEGY-37 | 1-4-22-23-36-9 | 139 | 4 | 2015 | Sputum | Egypt | N/A | M | 75 |
| CTEGY-38 | 1-7-1-6-52-4 | 237 | 2 | 2015 | Urine | Egypt | N/A | M | 67 |
| CTEGY-39 | 1-4-22-23-36-9 | 139 | 4 | 2015 | Urine | Egypt | N/A | M | 68 |
| CTEGY-40 | 1-42-1-7-54-1 | 1165 | 62 | 2015 | Urine | Egypt | N/A | F | 62 |
| CTEGY-41 | 1-32-22-23-36-9 | 1169 | 4 | 2015 | Blood | Egypt | N/A | M | 58 |
| CTEGY-42 | 10-195-3-42-205-44 | 1170 | S | 2015 | Blood | Egypt | N/A | F | 64 |
| CTEGY-44 | 1-7-1-6-52-4 | 237 | 2 | 2015 | Urine | Egypt | N/A | M | 53 |
| CTEGY-45 | 1-4-22-23-36-9 | 139 | 4 | 2015 | Urine | Egypt | N/A | M | 58 |
| CTEGY-49 | 1-6-3-5-5-3 | 6 | S | 2015 | Urine | Egypt | N/A | M | 61 |
| CTEGY-50 | 1-3-3-17-57-3 | 168 | 1 | 2015 | Urine | Egypt | N/A | M | 65 |
| CTEGY-51 | 1-92-1-10-1-3 | 1168 | S | 2015 | Urine | Egypt | N/A | M | 59 |
| CTEGY-52 | 1-9-22-23-36-9 | 401 | 4 | 2015 | Urine | Egypt | N/A | M | 90 |
| CTEGY-53 | 1-4-22-23-36-9 | 139 | 4 | 2015 | Urine | Egypt | N/A | M | 66 |
| CTEGY-54 | 1-4-22-23-36-9 | 139 | 4 | 2015 | ETT (Endotracheal Tube) | Egypt | N/A | M | 52 |
| CTEGY-55 | 3-7-3-6-52-4 | 1171 | 2 | 2015 | Urine | Egypt | N/A | M | 57 |
| CTEGY-56 | 1-9-22-23-36-9 | 401 | 4 | 2015 | Urine | Egypt | N/A | M | 62 |
| CTEGY-57 | 1-9-22-23-36-9 | 401 | 4 | 2015 | Sputum | Egypt | N/A | M | 74 |
| CTEGY-61 | 5-53-1-34-69-9 | 1172 | S | 2015 | Urine | Egypt | N/A | F | 54 |
| CTEGY-62 | 1-9-22-23-36-9 | 401 | 4 | 2015 | Urine | Egypt | N/A | M | 54 |
| CTEGY-63 | 1-7-3-10-76-6 | 1173 | 7 | 2015 | Urine | Egypt | N/A | M | 56 |
| CTEGY-65 | 1-32-22-23-36-9 | 1169 | 4 | 2015 | Urine | Egypt | N/A | F | 54 |
| CTEGY-67 | 1-9-22-23-36-9 | 401 | 4 | 2015 | Urine | Egypt | N/A | M | 62 |
| CTEGY-68 | 1-9-22-23-36-9 | 401 | 4 | 2015 | Urine | Egypt | N/A | M | 63 |
| CTEGY-69 | 1-4-22-23-36-9 | 139 | 4 | 2015 | Urine | Egypt | N/A | F | 57 |
| CTEGY-71 | 1-9-22-23-36-9 | 401 | 4 | 2015 | Sputum | Egypt | N/A | M | 69 |
| CTEGY-72 | 1-4-22-23-36-9 | 139 | 4 | 2015 | Urine | Egypt | N/A | M | 63 |
| CTEGY-73 | 1-196-3-7-76-4 | 1174 | S | 2015 | Urine | Egypt | N/A | F | 58 |
| CTEGY-74 | 1-7-3-10-73-6 | 1175 | 7 | 2015 | Urine | Egypt | N/A | M | 66 |
| CTCMR-2 | 1-7-3-7-73-6 | 1176 | 7 | 2013 | Vaginal | Cameroon | HIV+ | M | 45 |
| CTCMR-3 | 3-148-12-11-74-3 | 1186 | 63 | 2013 | Vaginal | Cameroon | HIV+ | F | 40 |
| CTCMR-4 | 3-148-12-11-74-3 | 1186 | 63 | 2013 | Vaginal | Cameroon | HIV+ | F | 40 |
| CTCMR-5 | 1-7-3-10-73-6 | 1175 | 7 | 2013 | Stool | Cameroon | HIV+ | F | 35 |
| CTCMR-6 | 1-76-1-10-73-40 | 1177 | S | 2013 | Urine | Cameroon | HIV+ | F | 32 |
| CTCMR-7 | 1-76-1-10-73-40 | 1177 | S | 2013 | Vaginal | Cameroon | HIV+ | F | 32 |
| CTCMR-9 | 1-3-3-11-175-6 | 1178 | S | 2013 | Urine | Cameroon | HIV+ | M | 55 |
| CTCMR-10 | 3-4-1-41-77-4 | 346 | 15 | 2013 | Vaginal | Cameroon | HIV+ | F | 35 |
| CTCMR-11 | 3-148-12-11-175-6 | 1195 | S | 2013 | Urine | Cameroon | HIV+ | M | 50 |
| CTCMR-12 | 1-3-3-11-175-6 | 1178 | S | 2013 | Urine | Cameroon | HIV+ | F | 57 |
| CTCMR-13 | 1-39-3-7-80-17 | 1179 | 64 | 2013 | Stool | Cameroon | HIV+ | M | 50 |
| CTCMR-14 | 1-44-1-7-58-3 | 522 | 6 | 2013 | Stool | Cameroon | HIV+ | M | 46 |
| CTCMR-15 | 1-39-3-7-80-17 | 1179 | 64 | 2013 | Stool | Cameroon | HIV+ | F | 43 |
| CTCMR-16 | 3-198-12-11-74-3 | 1196 | 63 | 2013 | Stool | Cameroon | HIV+ | F | 43 |
| CTCMR-17 | 1-26-1-7-206-3 | 1180 | S | 2013 | Stool | Cameroon | HIV+ | F | 33 |
| CTCMR-18 | 1-3-1-11-24-10 | 1181 | S | 2013 | Oral | Cameroon | HIV+ | F | 26 |
| CTCMR-19 | 1-44-1-7-58-3 | 522 | 6 | 2013 | Stool | Cameroon | HIV+ | F | 54 |
| CTCMR-20 | 1-44-1-7-58-6 | 1182 | 6 | 2013 | Stool | Cameroon | HIV+ | F | 54 |
| CTCMR-21 | 1-7-1-7-207-1 | 1183 | S | 2013 | Vaginal | Cameroon | HIV+ | F | 31 |
| CTCMR-40 | 1-39-3-7-80-9 | 1185 | 64 | 2014 | Stool | Cameroon | Diabetic | M | 58 |
| CTCMR-46 | 1-39-3-7-80-17 | 1179 | 64 | 2014 | Urine | Cameroon | Diabetic | F | 53 |
| CTCMR-47 | 1-197-1-10-48-1 | 1184 | S | 2014 | Vaginal | Cameroon | Diabetic | F | 50 |
| CTCMR-50 | 1-39-3-7-80-17 | 1179 | 64 | 2014 | Urine | Cameroon | Diabetic | M | 58 |
| CTCMR-51 | 1-39-3-7-80-17 | 1179 | 64 | 2014 | Stool | Cameroon | Diabetic | F | 46 |
| CTCMR-58 | 1-39-3-7-80-17 | 1179 | 64 | 2016 | Oral | Cameroon | Diabetic | M | 64 |
| CBS 2313 | 3-58-1-3-34-3 | 903 | S | 1930 | Superficial interdigital lesion | Egypt |  |  |  |
| CBS 2317 | 3-9-3-8-60-6 | 904 | 106 | N/A | Fodder | Russia |  |  |  |
| CBS 13074 | 1-6-3-5-5-3 | 6 | S | 1966 | Seawater | Antarctica |  |  |  |
| CBS 13075 | 10-19-60-101-159-58 | 905 | S | 1966 | Seawater | Antarctica |  |  |  |
| CBS 13076 | 1-6-3-5-5-3 | 6 | S | 1966 | Seawater | Antarctica |  |  |  |
| CBS 13077 | 10-19-60-101-159-58 | 905 | S | 1966 | Seawater | Antarctica |  |  |  |
| **Abbreviations: MLST, Multilocus Sequence Typing; DSTs, Diploid Sequence Types; CCs, Clonal complexes; N/A, Not available.** | | | | | | | | | |

| **Supplementary Table S1. Minimal inhibitory concentration (MIC) results obtained from the antifungal susceptibility tests (AFST) employed in this study. Clinical and Laboratory Standards Institute (CLSI) MIC breakpoints, interpretive categories, and epidemiological cut-off values (ECVs) are shown below.** | | | | | | | | | | | | | | | | | | |
| --- | --- | --- | --- | --- | --- | --- | --- | --- | --- | --- | --- | --- | --- | --- | --- | --- | --- | --- |
| **Isolate code** | **Amphotericin B** | | **Caspofungin** | | **Fluconazole** | | **Micafungin** | | **Flucytosine** | | **Voriconazole** | | **Anidulafungin** | | **Itraconazole** | | **Posaconazole** | |
|  | **MIC** | **Int** | **MIC** | **Int** | **MIC** | **Int** | **MIC** | **Int** | **MIC** | **Int** | **MIC** | **Int** | **MIC** | **Int** | **MIC** | **Int** | **MIC** | **Int** |
| CTRC-1 | 0,5 | WT | ≤ 0.25 | S | ≤ 1 | S | ≤ 0.06 | S | ≤ 1 |  | ≤ 0.12 | S |  |  |  |  |  |  |
| CTRC-2 | 0,5 | WT | ≤ 0.25 | S | ≤ 1 | S | ≤ 0.06 | S | ≤ 1 |  | ≤ 0.12 | S |  |  |  |  |  |  |
| CTRC-3 | 0,5 | WT | ≤ 0.25 | S | ≤ 1 | S | ≤ 0.06 | S | ≤ 1 |  | ≤ 0.12 | S |  |  |  |  |  |  |
| CTRC-4 | 0,5 | WT | ≤ 0.25 | S | ≤ 1 | S | ≤ 0.06 | S | ≤ 1 |  | ≤ 0.12 | S |  |  |  |  |  |  |
| CTRC-5 | 0,5 | WT | ≤ 0.25 | S | ≤ 1 | S | ≤ 0.06 | S | ≤ 1 |  | ≤ 0.12 | S |  |  |  |  |  |  |
| CTRC-6 | 0,5 | WT | ≤ 0.25 | S | ≤ 1 | S | ≤ 0.06 | S | ≤ 1 |  | ≤ 0.12 | S |  |  |  |  |  |  |
| CTRC-7 | 0,5 | WT | ≤ 0.25 | S | ≤ 1 | S | ≤ 0.06 | S | ≤ 1 |  | ≤ 0.12 | S |  |  |  |  |  |  |
| CTRC-8 | 0,5 | WT | ≤ 0.25 | S | ≤ 1 | S | ≤ 0.06 | S | ≤ 1 |  | ≤ 0.12 | S |  |  |  |  |  |  |
| CTRC-9 | 0,5 | WT | ≤ 0.25 | S | ≤ 1 | S | ≤ 0.06 | S | ≤ 1 |  | ≤ 0.12 | S |  |  |  |  |  |  |
| CTRC-10 | 0,5 | WT | ≤ 0.25 | S | ≤ 1 | S | ≤ 0.06 | S | ≤ 1 |  | ≤ 0.12 | S |  |  |  |  |  |  |
| CTRC-11 | 0,5 | WT | ≤ 0.25 | S | ≤ 1 | S | ≤ 0.06 | S | ≤ 1 |  | ≤ 0.12 | S |  |  |  |  |  |  |
| CTEGY-1 | 0,5 | WT | ≤ 0.25 | S | 16 | R | ≤ 0.06 | S | ≤ 1 |  | 0,5 | I |  |  |  |  |  |  |
| CTEGY-2 | 0,5 | WT | ≤ 0.25 | S | ≤ 1 | S | ≤ 0.06 | S | ≤ 1 |  | ≤ 0.12 | S |  |  |  |  |  |  |
| CTEGY-4 | 0,5 | WT | ≤ 0.25 | S | 32 | R | ≤ 0.06 | S | ≤ 1 |  | 0.5 | I |  |  |  |  |  |  |
| CTEGY-5 | ≤ 0.25 | WT | ≤ 0.25 | S | ≥ 64 | R | ≤ 0.06 | S | ≤ 1 |  | 1 | R |  |  |  |  |  |  |
| CTEGY-6 | 0,5 | WT | ≤ 0.25 | S | ≤ 1 | S | ≤ 0.06 | S | ≤ 1 |  | ≤ 0.12 | S |  |  |  |  |  |  |
| CTEGY-7 | 0,5 | WT | ≤ 0.25 | S | 32 | R | ≤ 0.06 | S | ≤ 1 |  | 0.5 | I |  |  |  |  |  |  |
| CTEGY-8 | 0,5 | WT | ≤ 0.25 | S | ≤ 1 | S | ≤ 0.06 | S | ≤ 1 |  | ≤ 0.12 | S |  |  |  |  |  |  |
| CTEGY-9 | 0,5 | WT | ≤ 0.25 | S | 8 | R | ≤ 0.06 | S | ≤ 1 |  | 0.25 | I |  |  |  |  |  |  |
| CTEGY-10 | 0.5 | WT | ≤ 0.25 | S | ≤ 1 | S | ≤ 0.06 | S | ≤ 1 |  | ≤ 0.12 | S |  |  |  |  |  |  |
| CTEGY-13 | 0.5 | WT | ≤ 0.25 | S | 8 | R | ≤ 0.06 | S | ≤ 1 |  | ≤ 0.12 | S |  |  |  |  |  |  |
| CTEGY-14 | 0.5 | WT | ≤ 0.25 | S | ≤ 1 | S | ≤ 0.06 | S | ≤ 1 |  | ≤ 0.12 | S |  |  |  |  |  |  |
| CTEGY-15 | 0.5 | WT | ≤ 0.25 | S | 32 | R | ≤ 0.06 | S | ≤ 1 |  | 0.5 | I |  |  |  |  |  |  |
| CTEGY-16 | 0.5 | WT | ≤ 0.25 | S | ≤ 1 | S | ≤ 0.06 | S | ≤ 1 |  | ≤ 0.12 | S |  |  |  |  |  |  |
| CTEGY-17 | 0.5 | WT | ≤ 0.25 | S | ≤ 1 | S | ≤ 0.06 | S | ≤ 1 |  | ≤ 0.12 | S |  |  |  |  |  |  |
| CTEGY-18 | 0.5 | WT | ≤ 0.25 | S | ≤ 1 | S | ≤ 0.06 | S | ≤ 1 |  | ≤ 0.12 | S |  |  |  |  |  |  |
| CTEGY-19 | 0.5 | WT | ≤ 0.25 | S | ≤ 1 | S | ≤ 0.06 | S | ≤ 1 |  | ≤ 0.12 | S |  |  |  |  |  |  |
| CTEGY-20 | ≤ 0.25 | WT | ≤ 0.25 | S | ≤ 1 | S | ≤ 0.06 | S | ≤ 1 |  | ≤ 0.12 | S |  |  |  |  |  |  |
| CTEGY-21 | 0.5 | WT | ≤ 0.25 | S | ≤ 1 | S | ≤ 0.06 | S | ≤ 1 |  | ≤ 0.12 | S |  |  |  |  |  |  |
| CTEGY-22 | 0.5 | WT | ≤ 0.25 | S | 4 | SDD | ≤ 0.06 | S | ≤ 1 |  | 0.25 | I |  |  |  |  |  |  |
| CTEGY-23 | ≤ 0.25 | WT | ≤ 0.25 | S | 8 | R | ≤ 0.06 | S | ≤ 1 |  | 0.25 | I |  |  |  |  |  |  |
| CTEGY-24 | 0.5 | WT | ≤ 0.25 | S | ≤ 1 | S | ≤ 0.06 | S | ≤ 1 |  | ≤ 0.12 | S |  |  |  |  |  |  |
| CTEGY-25 | ≤ 0.25 | WT | ≤ 0.25 | S | ≤ 1 | S | ≤ 0.06 | S | ≤ 1 |  | ≤ 0.12 | S |  |  |  |  |  |  |
| CTEGY-26 | 0.5 | WT | ≤ 0.25 | S | 8 | R | ≤ 0.06 | S | ≤ 1 |  | 0.25 | I |  |  |  |  |  |  |
| CTEGY-27 | ≤ 0.25 | WT | ≤ 0.25 | S | 4 | SDD | ≤ 0.06 | S | ≤ 1 |  | 0.25 | I |  |  |  |  |  |  |
| CTEGY-28 | 0.5 | WT | ≤ 0.25 | S | ≤ 1 | S | ≤ 0.06 | S | ≤ 1 |  | ≤ 0.12 | S |  |  |  |  |  |  |
| CTEGY-29 | ≤ 0.25 | WT | ≤ 0.25 | S | ≤ 1 | S | ≤ 0.06 | S | ≤ 1 |  | ≤ 0.12 | S |  |  |  |  |  |  |
| CTEGY-30 | ≤ 0.25 | WT | ≤ 0.25 | S | ≤ 1 | S | ≤ 0.06 | S | ≤ 1 |  | ≤ 0.12 | S |  |  |  |  |  |  |
| CTEGY-31 | ≤ 0.25 | WT | ≤ 0.25 | S | ≤ 1 | S | ≤ 0.06 | S | ≤ 1 |  | ≤ 0.12 | S |  |  |  |  |  |  |
| CTEGY-32 | 0.5 | WT | ≤ 0.25 | S | ≤ 1 | S | ≤ 0.06 | S | ≤ 1 |  | ≤ 0.12 | S |  |  |  |  |  |  |
| CTEGY-33 | 0.5 | WT | ≤ 0.25 | S | ≤ 1 | S | ≤ 0.06 | S | ≤ 1 |  | ≤ 0.12 | S |  |  |  |  |  |  |
| CTEGY-34 | ≤ 0.25 | WT | ≤ 0.25 | S | ≤ 1 | S | ≤ 0.06 | S | ≤ 1 |  | ≤ 0.12 | S |  |  |  |  |  |  |
| CTEGY-35 | 0.5 | WT | ≤ 0.25 | S | ≤ 1 | S | ≤ 0.06 | S | ≤ 1 |  | ≤ 0.12 | S |  |  |  |  |  |  |
| CTEGY-36 | 0.5 | WT | ≤ 0.25 | S | ≤ 1 | S | ≤ 0.06 | S | ≤ 1 |  | ≤ 0.12 | S |  |  |  |  |  |  |
| CTEGY-37 | ≤ 0.25 | WT | ≤ 0.25 | S | ≤ 1 | S | ≤ 0.06 | S | ≤ 1 |  | ≤ 0.12 | S |  |  |  |  |  |  |
| CTEGY-38 | 0.5 | WT | ≤ 0.25 | S | 8 | R | ≤ 0.06 | S | ≤ 1 |  | 0.25 | I |  |  |  |  |  |  |
| CTEGY-39 | 0.5 | WT | 2 | R | ≤ 1 | S | ≤ 0.06 | S | ≤ 1 |  | ≤ 0.12 | S |  |  |  |  |  |  |
| CTEGY-40 | 0.5 | WT | ≤ 0.25 | S | 8 | R | ≤ 0.06 | S | ≤ 1 |  | 0.25 | I |  |  |  |  |  |  |
| CTEGY-41 | 0.5 | WT | ≤ 0.25 | S | ≤ 1 | S | ≤ 0.06 | S | ≤ 1 |  | ≤ 0.12 | S |  |  |  |  |  |  |
| CTEGY-42 | 0.5 | WT | ≤ 0.25 | S | ≤ 1 | S | ≤ 0.06 | S | ≤ 1 |  | ≤ 0.12 | S |  |  |  |  |  |  |
| CTEGY-44 | ≤ 0.25 | WT | ≤ 0.25 | S | ≤ 1 | S | ≤ 0.06 | S | ≤ 1 |  | ≤ 0.12 | S |  |  |  |  |  |  |
| CTEGY-45 | ≤ 0.25 | WT | ≤ 0.25 | S | ≤ 1 | S | ≤ 0.06 | S | ≤ 1 |  | ≤ 0.12 | S |  |  |  |  |  |  |
| CTEGY-49 | ≤ 0.25 | WT | ≤ 0.25 | S | ≤ 1 | S | ≤ 0.06 | S | ≤ 1 |  | ≤ 0.12 | S |  |  |  |  |  |  |
| CTEGY-50 | 0.5 | WT | ≤ 0.25 | S | ≤ 1 | S | ≤ 0.06 | S | ≤ 1 |  | ≤ 0.12 | S |  |  |  |  |  |  |
| CTEGY-51 | 0.5 | WT | ≤ 0.25 | S | ≥ 64 | R | ≤ 0.06 | S | ≤ 1 |  | 1 | R |  |  |  |  |  |  |
| CTEGY-52 | 0.5 | WT | ≤ 0.25 | S | 32 | R | ≤ 0.06 | S | ≤ 1 |  | 0.5 | I |  |  |  |  |  |  |
| CTEGY-53 | 0.5 | WT | ≤ 0.25 | S | 4 | SDD | ≤ 0.06 | S | ≤ 1 |  | 0.25 | I |  |  |  |  |  |  |
| CTEGY-54 | 0.5 | WT | ≤ 0.25 | S | ≤ 1 | S | ≤ 0.06 | S | ≤ 1 |  | ≤ 0.12 | S |  |  |  |  |  |  |
| CTEGY-55 | 0.5 | WT | ≤ 0.25 | S | 8 | R | ≤ 0.06 | S | ≤ 1 |  | 0.25 | I |  |  |  |  |  |  |
| CTEGY-56 | 0.5 | WT | ≤ 0.25 | S | 8 | R | ≤ 0.06 | S | ≤ 1 |  | 0.25 | I |  |  |  |  |  |  |
| CTEGY-57 | 0.5 | WT | ≤ 0.25 | S | ≤ 1 | S | ≤ 0.06 | S | ≤ 1 |  | ≤ 0.12 | S |  |  |  |  |  |  |
| CTEGY-61 | ≤ 0.25 | WT | ≤ 0.25 | S | 8 | R | ≤ 0.06 | S | ≤ 1 |  | 0.25 | I |  |  |  |  |  |  |
| CTEGY-62 | 0.5 | WT | ≤ 0.25 | S | 8 | R | ≤ 0.06 | S | ≤ 1 |  | 0.5 | I |  |  |  |  |  |  |
| CTEGY-63 | 0.5 | WT | ≤ 0.25 | S | 32 | R | ≤ 0.06 | S | ≤ 1 |  | 2 | R |  |  |  |  |  |  |
| CTEGY-65 | ≤ 0.25 | WT | ≤ 0.25 | S | 32 | R | ≤ 0.06 | S | ≤ 1 |  | 0.5 | I |  |  |  |  |  |  |
| CTEGY-67 | 0.5 | WT | ≤ 0.25 | S | 32 | R | ≤ 0.06 | S | ≤ 1 |  | 1 | R |  |  |  |  |  |  |
| CTEGY-68 | ≤ 0.25 | WT | ≤ 0.25 | S | ≤ 1 | S | ≤ 0.06 | S | ≤ 1 |  | ≤ 0.12 | S |  |  |  |  |  |  |
| CTEGY-69 | 0.5 | WT | ≤ 0.25 | S | 32 | R | ≤ 0.06 | S | ≤ 1 |  | 2 | R |  |  |  |  |  |  |
| CTEGY-71 | 0.5 | WT | ≤ 0.25 | S | 32 | R | ≤ 0.06 | S | ≤ 1 |  | 0.5 | I |  |  |  |  |  |  |
| CTEGY-72 | ≤ 0.25 | WT | ≤ 0.25 | S | ≤ 1 | S | ≤ 0.06 | S | ≤ 1 |  | ≤ 0.12 | S |  |  |  |  |  |  |
| CTEGY-73 | 0.5 | WT | ≤ 0.25 | S | 32 | R | ≤ 0.06 | S | ≤ 1 |  | 2 | R |  |  |  |  |  |  |
| CTEGY-74 | 0.5 | WT | ≤ 0.25 | S | 8 | R | ≤ 0.06 | S | ≤ 1 |  | 0.25 | I |  |  |  |  |  |  |
| CTCMR-2 | 1 | WT | 1 | R | 64 | R | 0.5 | I | < 0.06 | WT | 0.25 | I | 0.03 | S | 2 | NWT | 1 | NWT |
| CTCMR-3 | 0.5 | WT | 0.25 | S | 8 | R | 0.03 | S | 0.12 | WT | 0.25 | I | 0.12 | S | 0.5 | WT | 1 | NWT |
| CTCMR-4 | 0.25 | WT | 0.5 | I | 64 | R | 0.12 | S | 16 | NWT | 0.5 | I | 0.12 | S | 1 | NWT | 2 | NWT |
| CTCMR-5 | 1 | WT | 0.03 | S | 8 | R | 0.03 | S | < 0.06 | WT | 0.25 | I | 0.015 | S | 0.5 | WT | 0.5 | NWT |
| CTCMR-6 | 0.25 | WT | 0.06 | S | 2 | S | 0.06 | S | 0.25 | WT | 0.25 | I | 0.016 | S | 0.25 | WT | 0.25 | NWT |
| CTCMR-7 | 0.25 | WT | 0.25 | S | 32 | R | 0.03 | S | 0.5 | WT | 0.5 | I | 0.03 | S | 1 | NWT | 2 | NWT |
| CTCMR-9 | 0.5 | WT | 0.03 | S | 4 | SDD | 0.015 | S | 0.12 | WT | 2 | R | 0.04 | S | 0.5 | WT | 2 | NWT |
| CTCMR-10 | 0.5 | WT | 0.03 | S | 4 | SDD | 0.06 | S | 0.12 | WT | 0.5 | I | 0.03 | S | 0.5 | WT | 1 | NWT |
| CTCMR-11 | 0.25 | WT | 0.03 | S | 16 | R | 0.015 | S | 0.12 | WT | 0.5 | I | 0.015 | S | 1 | NWT | 2 | NWT |
| CTCMR-12 | 0.5 | WT | 0.03 | S | 8 | R | 0.03 | S | 0.12 | WT | 0.5 | I | 0.03 | S | 0.25 | WT | < 0.008 | WT |
| CTCMR-13 | 0.25 | WT | 0.5 | I | 64 | R | 0.03 | S | < 0.06 | WT | 4 | R | 0.5 | I | 1 | NWT | 2 | NWT |
| CTCMR-14 | 0.5 | WT | 0.5 | I | 32 | R | 0.03 | S | < 0.06 | WT | 1 | R | 0.06 | S | 1 | NWT | 2 | NWT |
| CTCMR-15 | 0.5 | WT | 0.03 | S | 16 | R | 0.03 | S | 0.12 | WT | 1 | R | 0.12 | S | 1 | NWT | 2 | NWT |
| CTCMR-16 | 0.25 | WT | 0.06 | S | 16 | R | 0.03 | S | < 0.06 | WT | 0.5 | I | 0.015 | S | 2 | NWT | 1 | NWT |
| CTCMR-17 | 1 | WT | 0.06 | S | 2 | S | 0.015 | S | 0.12 | WT | 0.12 | S | 0.12 | S | 0.25 | WT | 0.25 | NWT |
| CTCMR-18 | 0.25 | WT | 0.015 | S | 2 | S | 0.03 | S | < 0.06 | WT | 0.25 | I | 0.25 | S | 0.25 | WT | 0.5 | NWT |
| CTCMR-19 | 2 | WT | 0.03 | S | 2 | S | 0.015 | S | < 0.06 | WT | 0.12 | S | 0.015 | S | 0.12 | WT | 0.12 | WT |
| CTCMR-20 | 1 | WT | 0.015 | S | 32 | R | 0.015 | S | < 0.06 | WT | 2 | R | 0.03 | S | 1 | NWT | 2 | NWT |
| CTCMR-21 | 1 | WT | 0.12 | S | 32 | R | 0.015 | S | 0.12 | WT | 0.5 | I | 0.12 | S | 1 | NWT | 2 | NWT |
| CTCMR-40 | < 0.12 | WT | 0.06 | S | 2 | S | 0.015 | S | 0.12 | WT | 0.25 | I | 0.015 | S | 0.25 | WT | < 0.008 | WT |
| CTCMR-46 | 1 | WT | 0.03 | S | 2 | S | 0.03 | S | 0.12 | WT | 0.12 | S | 0.015 | S | 0.25 | WT | 0.5 | NWT |
| CTCMR-47 | < 0.12 | WT | 1 | R | 0.5 | S | 0.5 | I | 0.12 | WT | 0.06 | S | 0.5 | I | 0.12 | WT | 0.12 | WT |
| CTCMR-50 | 1 | WT | 0.25 | S | 2 | S | 1 | R | 0.12 | WT | 0.12 | S | 0.12 | S | 0.25 | WT | 0.25 | NWT |
| CTCMR-51 | 0.25 | WT | 0.03 | S | 32 | R | 0.015 | S | < 0.06 | WT | 2 | R | 0.06 | S | 1 | NWT | 2 | NWT |
| CTCMR-58 | 1 | WT | 0.03 | S | 2 | S | 0.03 | S | 0.12 | WT | 0.12 | S | 0.015 | S | 0.25 | WT | 0.5 | NWT |
| CBS 2313 |  |  |  |  |  |  |  |  |  |  |  |  |  |  |  |  |  |  |
| CBS 2317 |  |  |  |  |  |  |  |  |  |  |  |  |  |  |  |  |  |  |
| CBS 13074 |  |  |  |  |  |  |  |  |  |  |  |  |  |  |  |  |  |  |
| CBS 13075 |  |  |  |  |  |  |  |  |  |  |  |  |  |  |  |  |  |  |
| CBS 13076 |  |  |  |  |  |  |  |  |  |  |  |  |  |  |  |  |  |  |
| CBS 13077 |  |  |  |  |  |  |  |  |  |  |  |  |  |  |  |  |  |  |
| **Abbreviations: Int, Interpretation; MIC, Minimal Inhibitory Concentration; R, Resistant; S, Susceptible; SDD, Susceptible-Dose Dependent;: I, Intermediate; WT, Wild Type; NWT, Non Wild Type.** | | | | | | | | | | | | | | | | | | |

| **Antifungal drug** | **CLSI MIC Breakpoints and Interpretive Categories, μg/mL** | | | | **ECVs*, μg/mL** |
| --- | --- | --- | --- | --- | --- |
|  | **S*** | **I *** | **SDD*** | **R*** |  |
| Anidulafungin | ≤ 0.25 | 0.5 | -- | ≥ 1 | -- |
| Micafungin | ≤ 0.25 | 0.5 | -- | ≥ 1 | -- |
| Caspofungin | ≤ 0.25 | 0.5 | -- | ≥ 1 | -- |
| Flucytosine | -- | -- | -- | -- | 0,5 |
| Posaconazole | -- | -- | -- | -- | 0.12 |
| Voriconazole | ≤ 0.12 | 0.25–0.5 | -- | ≥ 1 | -- |
| Itraconazole | -- | -- | -- | -- | 0.5 |
| Fluconazole | ≤ 2 | -- | 4 | ≥ 8 | -- |
| Amphotericin B | -- | -- | -- | -- | 2 |
| ***Abbreviations: S, Susceptible; I, Intermediate; SDD, Susceptible-Dose Dependent; R, Resistant; ECVs, Epidemiological Cut-off Values.** | | | | | |
