# Supplementary material for: Multilocus sequence typing (MLST) analysis reveals many novel genotypes and a high level of genetic diversity in Candida tropicalis isolates from Italy and Africa
Source: Mycoses. 2022 Jul 7;65(11):989–1000. doi: 10.1111/myc.13483 (PMC9796097; doi:10.1111/myc.13483)
Supplement: Supplementary file 2 — Table S2 [file MYC-65-989-s003.docx]

**CC1**

| **Isolate n°** | **Country** | **Continent** | **MLST loci** | | | | | | **DST** | **CC** |
| --- | --- | --- | --- | --- | --- | --- | --- | --- | --- | --- |
|  |  |  | ***ICL1*** | ***MDR1*** | ***SAPT2*** | ***SAPT4*** | ***XYR1*** | ***ZWF1a*** |  |  |
| J990332/2 | USA | North America | 1 | 22 | 3 | 10 | 9 | 3 | 39 | 1 |
| YM060100 | Taiwan | Asia | 1 | 3 | 3 | 17 | 16 | 3 | 45 | 1 |
| b30980/5/04 | UK | Europe | 1 | 3 | 3 | 17 | 16 | 3 | 45 | 1 |
| AM2003/0078 | UK | Europe | 1 | 3 | 3 | 17 | 16 | 3 | 45 | 1 |
| AM2005/0272 | UK | Europe | 1 | 3 | 3 | 17 | 9 | 7 | 97 | 1 |
| YM990131 | Taiwan | Asia | 1 | 3 | 3 | 17 | 9 | 3 | 98 | 1 |
| YM990135 | Taiwan | Asia | 1 | 3 | 3 | 17 | 9 | 3 | 98 | 1 |
| YM060450 | Taiwan | Asia | 1 | 3 | 3 | 17 | 9 | 3 | 98 | 1 |
| YM060451 | Taiwan | Asia | 1 | 3 | 3 | 17 | 9 | 3 | 98 | 1 |
| YM060647 | Taiwan | Asia | 1 | 3 | 3 | 17 | 9 | 3 | 98 | 1 |
| YM060529 | Taiwan | Asia | 1 | 3 | 3 | 17 | 9 | 3 | 98 | 1 |
| YM060547 | Taiwan | Asia | 1 | 3 | 3 | 17 | 9 | 3 | 98 | 1 |
| AM2005/0089 | Colombia | South America | 1 | 3 | 3 | 17 | 9 | 3 | 98 | 1 |
| AM2005/0091 | Colombia | South America | 1 | 3 | 3 | 17 | 9 | 3 | 98 | 1 |
| YM990145 | Taiwan | Asia | 1 | 7 | 3 | 17 | 9 | 1 | 136 | 1 |
| YM990148 | Taiwan | Asia | 1 | 3 | 24 | 17 | 54 | 3 | 137 | 1 |
| CTEGY-13 | Egypt | Africa | 1 | 3 | 3 | 17 | 54 | 3 | 140 | 1 |
| YM060327 | Taiwan | Asia | 1 | 3 | 3 | 17 | 54 | 3 | 140 | 1 |
| YM990490 | Taiwan | Asia | 1 | 3 | 3 | 17 | 54 | 3 | 140 | 1 |
| YM020309 | Taiwan | Asia | 1 | 3 | 3 | 17 | 54 | 3 | 140 | 1 |
| YM990603 | Taiwan | Asia | 1 | 3 | 3 | 17 | 54 | 3 | 140 | 1 |
| YM990592 | Taiwan | Asia | 1 | 3 | 3 | 17 | 54 | 3 | 140 | 1 |
| YM020919 | Taiwan | Asia | 1 | 3 | 3 | 17 | 54 | 3 | 140 | 1 |
| YM990645 | Taiwan | Asia | 1 | 3 | 3 | 17 | 54 | 3 | 140 | 1 |
| YM990647 | Taiwan | Asia | 1 | 3 | 3 | 17 | 54 | 3 | 140 | 1 |
| YM990649 | Taiwan | Asia | 1 | 3 | 3 | 17 | 54 | 3 | 140 | 1 |
| YM990659 | Taiwan | Asia | 1 | 3 | 3 | 17 | 54 | 3 | 140 | 1 |
| YM990660 | Taiwan | Asia | 1 | 3 | 3 | 17 | 54 | 3 | 140 | 1 |
| YM990662 | Taiwan | Asia | 1 | 3 | 3 | 17 | 54 | 3 | 140 | 1 |
| YM060509 | Taiwan | Asia | 1 | 3 | 3 | 17 | 54 | 3 | 140 | 1 |
| YM990275 | Taiwan | Asia | 1 | 3 | 3 | 17 | 54 | 3 | 140 | 1 |
| YM060098 | Taiwan | Asia | 1 | 3 | 3 | 17 | 54 | 3 | 140 | 1 |
| DOH38 | Taiwan | Asia | 1 | 3 | 3 | 17 | 54 | 3 | 140 | 1 |
| YM060102 | Taiwan | Asia | 1 | 3 | 3 | 17 | 54 | 3 | 140 | 1 |
| YM060173 | Taiwan | Asia | 1 | 3 | 3 | 17 | 54 | 3 | 140 | 1 |
| YM990577 | Taiwan | Asia | 1 | 3 | 3 | 17 | 54 | 3 | 140 | 1 |
| YM060828 | Taiwan | Asia | 1 | 3 | 3 | 17 | 54 | 3 | 140 | 1 |
| YM990537 | Taiwan | Asia | 1 | 3 | 3 | 17 | 54 | 3 | 140 | 1 |
| YM020273 | Taiwan | Asia | 1 | 3 | 3 | 17 | 54 | 3 | 140 | 1 |
| NHUE10 | Taiwan | Asia | 1 | 3 | 3 | 17 | 54 | 3 | 140 | 1 |
| NHUE48 | Taiwan | Asia | 1 | 3 | 3 | 17 | 54 | 3 | 140 | 1 |
| NHUE56 | Taiwan | Asia | 1 | 3 | 3 | 17 | 54 | 3 | 140 | 1 |
| YM990458 | Taiwan | Asia | 1 | 3 | 3 | 17 | 54 | 1 | 144 | 1 |
| YM020294 | Taiwan | Asia | 1 | 3 | 3 | 17 | 54 | 1 | 144 | 1 |
| YM990554 | Taiwan | Asia | 1 | 7 | 3 | 17 | 57 | 3 | 147 | 1 |
| CTEGY-50 | Egypt | Africa | 1 | 3 | 3 | 17 | 57 | 3 | 168 | 1 |
| DOH60 | Taiwan | Asia | 1 | 3 | 3 | 17 | 57 | 3 | 168 | 1 |
| DOH73 | Taiwan | Asia | 1 | 3 | 3 | 17 | 57 | 3 | 168 | 1 |
| YM060136 | Taiwan | Asia | 1 | 3 | 3 | 17 | 57 | 3 | 168 | 1 |
| NHUE23 | Taiwan | Asia | 1 | 3 | 3 | 17 | 57 | 3 | 168 | 1 |
| YM060776 | Taiwan | Asia | 1 | 3 | 3 | 17 | 54 | 7 | 179 | 1 |
| YM060175 | Taiwan | Asia | 1 | 3 | 3 | 17 | 54 | 7 | 179 | 1 |
| YM060144 | Taiwan | Asia | 1 | 61 | 3 | 17 | 54 | 3 | 180 | 1 |
| YM060590 | Taiwan | Asia | 1 | 3 | 3 | 17 | 54 | 23 | 181 | 1 |
| YM060805 | Taiwan | Asia | 1 | 3 | 3 | 17 | 54 | 24 | 182 | 1 |
| NHUE27 | Taiwan | Asia | 1 | 81 | 3 | 17 | 57 | 3 | 229 | 1 |
| BZR-2 | China | Asia | 1 | 3 | 3 | 17 | 16 | 7 | 269 | 1 |
| BZR-75 | China | Asia | 1 | 3 | 3 | 7 | 16 | 3 | 297 | 1 |
| F2011z016 | Taiwan | Asia | 1 | 3 | 3 | 17 | 99 | 3 | 357 | 1 |
| F2011x051 | Taiwan | Asia | 1 | 3 | 3 | 17 | 9 | 1 | 383 | 1 |
| F2013a014 | Taiwan | Asia | 1 | 3 | 3 | 17 | 9 | 23 | 388 | 1 |
| F2013a087 | Taiwan | Asia | 1 | 98 | 3 | 17 | 54 | 3 | 389 | 1 |
| SM11 | South Korea | Asia | 1 | 3 | 3 | 17 | 106 | 3 | 406 | 1 |
| 27_HNHK_M_52_PT | China | Asia | 1 | 3 | 3 | 10 | 3 | 3 | 416 | 1 |
| F2011a001 | Taiwan | Asia | 1 | 3 | 3 | 17 | 122 | 3 | 548 | 1 |
| F2014e100-2 | Taiwan | Asia | 1 | 32 | 3 | 17 | 54 | 3 | 560 | 1 |
| YM140212 | Taiwan | Asia | 1 | 3 | 3 | 17 | 43 | 1 | 572 | 1 |
| 8638 | China | Asia | 1 | 3 | 3 | 10 | 9 | 3 | 726 | 1 |
| IRCCS 28 | Italy | Europe | 1 | 7 | 3 | 10 | 9 | 3 | 751 | 1 |
| IRCCS 29 | Italy | Europe | 1 | 7 | 3 | 10 | 9 | 3 | 751 | 1 |
| C2-1020619 | Taiwan | Asia | 1 | 32 | 3 | 17 | 54 | 50 | 790 | 1 |
| C2-1030117 | Taiwan | Asia | 43 | 3 | 3 | 17 | 16 | 3 | 794 | 1 |
| C2-1030507 | Taiwan | Asia | 1 | 159 | 3 | 17 | 9 | 3 | 795 | 1 |
| C2-1030706 | Taiwan | Asia | 1 | 3 | 3 | 89 | 54 | 3 | 797 | 1 |
| C2-1031004 | Taiwan | Asia | 1 | 160 | 3 | 17 | 54 | 3 | 798 | 1 |
| F2017a060 | Taiwan | Asia | 1 | 152 | 3 | 17 | 9 | 3 | 820 | 1 |
| F2017c051 | Taiwan | Asia | 1 | 3 | 3 | 17 | 150 | 3 | 829 | 1 |
| F2017c080 | Taiwan | Asia | 1 | 3 | 3 | 17 | 151 | 3 | 830 | 1 |
| C2-1000504 | Taiwan | Asia | 1 | 3 | 3 | 7 | 54 | 3 | 840 | 1 |
| C2-1020612 | Taiwan | Asia | 1 | 3 | 3 | 10 | 48 | 3 | 845 | 1 |
| F2013f085 | Taiwan | Asia | 1 | 3 | 3 | 14 | 54 | 3 | 858 | 1 |
| F2016g026 | Taiwan | Asia | 1 | 3 | 3 | 14 | 16 | 3 | 859 | 1 |
| F2016d044 | Taiwan | Asia | 1 | 32 | 3 | 14 | 54 | 3 | 871 | 1 |
| F2016d098 | Taiwan | Asia | 3 | 3 | 3 | 17 | 54 | 3 | 885 | 1 |
| YM140896 | Taiwan | Asia | 1 | 7 | 3 | 17 | 54 | 3 | 911 | 1 |
| CTRC-05 | Italy | Europe | 1 | 7 | 3 | 17 | 54 | 3 | 911 | 1 |
| CTRC-06 | Italy | Europe | 1 | 7 | 3 | 17 | 54 | 3 | 911 | 1 |
| T_Y480 | South Korea | Asia | 1 | 3 | 3 | 56 | 9 | 3 | 928 | 1 |
| T_Y359 | South Korea | Asia | 1 | 3 | 3 | 56 | 57 | 3 | 929 | 1 |
| YM180358 | Taiwan | Asia | 1 | 3 | 3 | 17 | 54 | 22 | 953 | 1 |
| YM180404 | Taiwan | Asia | 1 | 3 | 3 | 104 | 54 | 3 | 954 | 1 |
| YM180556 | Taiwan | Asia | 1 | 3 | 61 | 17 | 54 | 3 | 955 | 1 |
| CW0071 | Singapore | Asia | 1 | 3 | 3 | 14 | 57 | 7 | 1005 | 1 |
| CS0424 | Singapore | Asia | 1 | 3 | 3 | 111 | 57 | 3 | 1006 | 1 |
| CS0943 | Singapore | Asia | 1 | 3 | 3 | 113 | 57 | 3 | 1007 | 1 |
| CW0385 | Singapore | Asia | 1 | 3 | 3 | 113 | 57 | 7 | 1008 | 1 |
| CS1573 | Singapore | Asia | 1 | 3 | 3 | 113 | 92 | 3 | 1009 | 1 |
| CS1178 | Singapore | Asia | 1 | 3 | 3 | 116 | 57 | 7 | 1011 | 1 |
| F2019i068 | Taiwan | Asia | 1 | 3 | 3 | 14 | 9 | 3 | 1111 | 1 |
| **Abbreviations: MLST: Multi Locus Sequence Typing; DST: Diploid Sequence Type; CC: Clonal Complex.** | | | | | | | | | | |

**CC2**

| **Isolate n°** | **Country** | **Continent** | **MLST loci** | | | | | | **DST** | **CC** |
| --- | --- | --- | --- | --- | --- | --- | --- | --- | --- | --- |
|  |  |  | ***ICL1*** | ***MDR1*** | ***SAPT2*** | ***SAPT4*** | ***XYR1*** | ***ZWF1a*** |  |  |
| J980160 | USA | North America | 3 | 7 | 3 | 6 | 6 | 4 | 7 | 2 |
| J980156 | USA | North America | 3 | 7 | 3 | 6 | 6 | 4 | 7 | 2 |
| A427748 | UK | Europe | 3 | 7 | 1 | 6 | 6 | 7 | 14 | 2 |
| YM060481 | Taiwan | Asia | 3 | 7 | 1 | 6 | 6 | 4 | 27 | 2 |
| NCCLS71 | USA | North America | 3 | 7 | 1 | 6 | 6 | 4 | 27 | 2 |
| J980157 | USA | North America | 3 | 7 | 3 | 15 | 6 | 4 | 30 | 2 |
| J980162 | USA | North America | 3 | 7 | 3 | 6 | 11 | 4 | 37 | 2 |
| b31597/6/04 | UK | Europe | 10 | 7 | 3 | 6 | 11 | 4 | 62 | 2 |
| b30488/7/04 | UK | Europe | 1 | 7 | 1 | 6 | 22 | 4 | 64 | 2 |
| YM990598 | Taiwan | Asia | 3 | 7 | 1 | 6 | 22 | 4 | 90 | 2 |
| YM020311 | Taiwan | Asia | 3 | 7 | 1 | 6 | 22 | 4 | 90 | 2 |
| YM020693 | Taiwan | Asia | 3 | 7 | 1 | 6 | 22 | 4 | 90 | 2 |
| GUI3622 | Brazil | South America | 3 | 7 | 1 | 6 | 22 | 4 | 90 | 2 |
| GUI3629A | Brazil | South America | 3 | 7 | 1 | 6 | 22 | 4 | 90 | 2 |
| GUI3719 | Brazil | South America | 3 | 7 | 1 | 6 | 22 | 4 | 90 | 2 |
| AM2005/0087 | Colombia | South America | 3 | 7 | 1 | 6 | 22 | 4 | 90 | 2 |
| AM2005/0088 | Colombia | South America | 3 | 7 | 1 | 6 | 22 | 4 | 90 | 2 |
| AM2005/0276 | UK | Europe | 1 | 7 | 1 | 6 | 6 | 4 | 106 | 2 |
| AM2005/0277 | UK | Europe | 1 | 7 | 1 | 6 | 6 | 4 | 106 | 2 |
| AM2005/0296 | UK | Europe | 1 | 7 | 1 | 6 | 6 | 4 | 106 | 2 |
| YM060507 | Taiwan | Asia | 3 | 7 | 1 | 6 | 52 | 4 | 134 | 2 |
| YM020743 | Taiwan | Asia | 3 | 7 | 1 | 6 | 52 | 4 | 134 | 2 |
| DOH81 | Taiwan | Asia | 3 | 7 | 1 | 6 | 52 | 4 | 134 | 2 |
| YM060299 | Taiwan | Asia | 3 | 7 | 1 | 6 | 52 | 4 | 134 | 2 |
| YM060508 | Taiwan | Asia | 3 | 7 | 1 | 6 | 52 | 4 | 134 | 2 |
| YM060512 | Taiwan | Asia | 3 | 7 | 1 | 6 | 52 | 4 | 134 | 2 |
| YM990138 | Taiwan | Asia | 3 | 7 | 1 | 6 | 52 | 4 | 134 | 2 |
| YM990533 | Taiwan | Asia | 3 | 7 | 1 | 6 | 2 | 4 | 145 | 2 |
| YM020274 | Taiwan | Asia | 3 | 7 | 1 | 6 | 60 | 4 | 153 | 2 |
| YM020291 | Taiwan | Asia | 3 | 7 | 3 | 6 | 22 | 4 | 155 | 2 |
| DOH7 | Taiwan | Asia | 3 | 7 | 1 | 6 | 54 | 4 | 175 | 2 |
| DOH78 | Taiwan | Asia | 3 | 7 | 1 | 6 | 67 | 4 | 178 | 2 |
| YM060379 | Taiwan | Asia | 3 | 65 | 1 | 6 | 52 | 4 | 200 | 2 |
| YM060800 | Taiwan | Asia | 3 | 65 | 1 | 6 | 52 | 4 | 200 | 2 |
| YM060325 | Taiwan | Asia | 3 | 7 | 4 | 6 | 75 | 10 | 201 | 2 |
| HY8_hn_m_75_Pt | China | Asia | 3 | 7 | 4 | 6 | 52 | 4 | 203 | 2 |
| T11 | India | Asia | 3 | 7 | 4 | 44 | 52 | 4 | 220 | 2 |
| T22 | India | Asia | 3 | 7 | 4 | 44 | 52 | 4 | 220 | 2 |
| T37 | India | Asia | 3 | 7 | 4 | 6 | 52 | 7 | 221 | 2 |
| T14 | India | Asia | 10 | 7 | 3 | 6 | 84 | 4 | 222 | 2 |
| T42 | India | Asia | 10 | 7 | 3 | 6 | 52 | 4 | 223 | 2 |
| T41 | India | Asia | 10 | 7 | 3 | 45 | 52 | 4 | 224 | 2 |
| CTEGY-35 | Egypt | Africa | 1 | 7 | 1 | 6 | 52 | 4 | 237 | 2 |
| CTEGY-38 | Egypt | Africa | 1 | 7 | 1 | 6 | 52 | 4 | 237 | 2 |
| CTEGY-44 | Egypt | Africa | 1 | 7 | 1 | 6 | 52 | 4 | 237 | 2 |
| 8a | Brazil | South America | 1 | 7 | 1 | 6 | 52 | 4 | 237 | 2 |
| 12a | Brazil | South America | 1 | 7 | 4 | 6 | 52 | 4 | 238 | 2 |
| 7b | Brazil | South America | 1 | 7 | 4 | 6 | 52 | 4 | 238 | 2 |
| 8b | Brazil | South America | 1 | 7 | 1 | 6 | 84 | 4 | 243 | 2 |
| 13a | Brazil | South America | 3 | 7 | 4 | 6 | 84 | 4 | 245 | 2 |
| 12b | Brazil | South America | 1 | 7 | 4 | 6 | 84 | 4 | 248 | 2 |
| F2013a053 | Taiwan | Asia | 34 | 7 | 1 | 6 | 52 | 4 | 377 | 2 |
| F2011aa040 | Taiwan | Asia | 3 | 7 | 1 | 6 | 52 | 10 | 384 | 2 |
| AJ71 | South Korea | Asia | 3 | 7 | 1 | 6 | 51 | 4 | 392 | 2 |
| C26_HNSY_M_17_HP | China | Asia | 3 | 7 | 1 | 6 | 52 | 3 | 484 | 2 |
| F2014c063-1 | Taiwan | Asia | 1 | 7 | 1 | 6 | 52 | 10 | 563 | 2 |
| YM140717 | Taiwan | Asia | 3 | 7 | 1 | 6 | 84 | 4 | 589 | 2 |
| CTEGY-17 | Egypt | Africa | 1 | 7 | 3 | 6 | 52 | 4 | 682 | 2 |
| CTEGY-21 | Egypt | Africa | 1 | 7 | 3 | 6 | 52 | 4 | 682 | 2 |
| CT183 | Taiwan | Asia | 3 | 7 | 54 | 6 | 60 | 4 | 778 | 2 |
| CT205 | Taiwan | Asia | 3 | 7 | 1 | 6 | 144 | 4 | 779 | 2 |
| CT262 | Taiwan | Asia | 1 | 7 | 1 | 6 | 145 | 4 | 780 | 2 |
| C2-1030116 | Taiwan | Asia | 3 | 7 | 1 | 6 | 145 | 4 | 793 | 2 |
| C2-1031201 | Taiwan | Asia | 3 | 161 | 1 | 6 | 52 | 4 | 799 | 2 |
| C2-1021205 | Taiwan | Asia | 3 | 7 | 1 | 6 | 149 | 4 | 802 | 2 |
| C2-1011006 | Taiwan | Asia | 3 | 7 | 4 | 6 | 67 | 4 | 807 | 2 |
| F2017b003 | Taiwan | Asia | 3 | 7 | 4 | 91 | 52 | 4 | 821 | 2 |
| F2017b015 | Taiwan | Asia | 3 | 7 | 1 | 91 | 52 | 4 | 875 | 2 |
| F2015f021 | Taiwan | Asia | 1 | 7 | 4 | 91 | 52 | 4 | 884 | 2 |
| T_Y696 | South Korea | Asia | 3 | 7 | 1 | 6 | 11 | 4 | 941 | 2 |
| CS0060 | Singapore | Asia | 3 | 7 | 1 | 6 | 186 | 4 | 1040 | 2 |
| CS0923 | Singapore | Asia | 3 | 7 | 1 | 6 | 186 | 4 | 1040 | 2 |
| F2016b002 | Taiwan | Asia | 3 | 7 | 4 | 6 | 67 | 10 | 1085 | 2 |
| CTEGY-26 | Egypt | Africa | 1 | 7 | 1 | 6 | 144 | 4 | 1166 | 2 |
| CTEGY-55 | Egypt | Africa | 3 | 7 | 3 | 6 | 52 | 4 | 1171 | 2 |
| IFM54674 | Japan | Asia | 3 | 7 | 69 | 6 | 52 | 4 | 1199 | 2 |
| **Abbreviations: MLST: Multi Locus Sequence Typing; DST: Diploid Sequence Type; CC: Clonal Complex.** | | | | | | | | | | |

**CC4**

| **Isolate n°** | **Country** | **Continent** | **MLST loci** | | | | | | **DST** | **CC** |
| --- | --- | --- | --- | --- | --- | --- | --- | --- | --- | --- |
|  |  |  | ***ICL1*** | ***MDR1*** | ***SAPT2*** | ***SAPT4*** | ***XYR1*** | ***ZWF1a*** |  |  |
| GUI4448 | Brazil | South America | 1 | 41 | 12 | 23 | 36 | 9 | 91 | 4 |
| 10H1048 | China | Asia | 1 | 4 | 22 | 23 | 43 | 9 | 114 | 4 |
| 8618 | China | Asia | 1 | 4 | 22 | 23 | 43 | 9 | 114 | 4 |
| AM2005/0549 | UK | Europe | 1 | 4 | 22 | 23 | 43 | 9 | 114 | 4 |
| AM2005/0556 | UK | Europe | 1 | 4 | 22 | 23 | 43 | 9 | 114 | 4 |
| AM2005/0557 | UK | Europe | 1 | 4 | 22 | 23 | 43 | 9 | 114 | 4 |
| YM990236 | Taiwan | Asia | 1 | 4 | 22 | 23 | 55 | 9 | 138 | 4 |
| CTEGY-02 | Egypt | Africa | 1 | 4 | 22 | 23 | 36 | 9 | 139 | 4 |
| CTEGY-04 | Egypt | Africa | 1 | 4 | 22 | 23 | 36 | 9 | 139 | 4 |
| CTEGY-05 | Egypt | Africa | 1 | 4 | 22 | 23 | 36 | 9 | 139 | 4 |
| CTEGY-15 | Egypt | Africa | 1 | 4 | 22 | 23 | 36 | 9 | 139 | 4 |
| CTEGY-16 | Egypt | Africa | 1 | 4 | 22 | 23 | 36 | 9 | 139 | 4 |
| CTEGY-19 | Egypt | Africa | 1 | 4 | 22 | 23 | 36 | 9 | 139 | 4 |
| CTEGY-23 | Egypt | Africa | 1 | 4 | 22 | 23 | 36 | 9 | 139 | 4 |
| CTEGY-27 | Egypt | Africa | 1 | 4 | 22 | 23 | 36 | 9 | 139 | 4 |
| CTEGY-33 | Egypt | Africa | 1 | 4 | 22 | 23 | 36 | 9 | 139 | 4 |
| CTEGY-34 | Egypt | Africa | 1 | 4 | 22 | 23 | 36 | 9 | 139 | 4 |
| CTEGY-37 | Egypt | Africa | 1 | 4 | 22 | 23 | 36 | 9 | 139 | 4 |
| CTEGY-39 | Egypt | Africa | 1 | 4 | 22 | 23 | 36 | 9 | 139 | 4 |
| CTEGY-45 | Egypt | Africa | 1 | 4 | 22 | 23 | 36 | 9 | 139 | 4 |
| CTEGY-53 | Egypt | Africa | 1 | 4 | 22 | 23 | 36 | 9 | 139 | 4 |
| CTEGY-54 | Egypt | Africa | 1 | 4 | 22 | 23 | 36 | 9 | 139 | 4 |
| CTEGY-69 | Egypt | Africa | 1 | 4 | 22 | 23 | 36 | 9 | 139 | 4 |
| CTEGY-72 | Egypt | Africa | 1 | 4 | 22 | 23 | 36 | 9 | 139 | 4 |
| E230 | China | Asia | 1 | 4 | 22 | 23 | 36 | 9 | 139 | 4 |
| YM990268 | Taiwan | Asia | 1 | 4 | 22 | 23 | 36 | 9 | 139 | 4 |
| YM060369 | Taiwan | Asia | 1 | 4 | 22 | 23 | 36 | 9 | 139 | 4 |
| NHUE42 | Taiwan | Asia | 1 | 4 | 22 | 23 | 36 | 9 | 139 | 4 |
| CTRC-11 | Italy | Europe | 1 | 4 | 22 | 23 | 36 | 9 | 139 | 4 |
| DOH102 | Taiwan | Asia | 1 | 4 | 3 | 23 | 36 | 9 | 171 | 4 |
| CTEGY-30 | Egypt | Africa | 1 | 4 | 12 | 23 | 36 | 9 | 184 | 4 |
| YM060210 | Taiwan | Asia | 1 | 4 | 12 | 23 | 36 | 9 | 184 | 4 |
| NHUE36 | Taiwan | Asia | 1 | 4 | 12 | 10 | 3 | 9 | 230 | 4 |
| F2011ah032 | Taiwan | Asia | 1 | 9 | 3 | 23 | 36 | 9 | 257 | 4 |
| BZR-25 | China | Asia | 1 | 4 | 22 | 23 | 85 | 9 | 276 | 4 |
| BZR-31 | China | Asia | 1 | 4 | 22 | 23 | 85 | 1 | 277 | 4 |
| BZR-78 | China | Asia | 1 | 4 | 22 | 23 | 85 | 1 | 277 | 4 |
| BZR-97 | China | Asia | 1 | 4 | 22 | 23 | 85 | 2 | 309 | 4 |
| 09H1006 | China | Asia | 1 | 4 | 12 | 23 | 43 | 9 | 321 | 4 |
| 09HX016 | China | Asia | 1 | 4 | 12 | 23 | 43 | 9 | 321 | 4 |
| 2128 | China | Asia | 1 | 4 | 12 | 23 | 43 | 9 | 321 | 4 |
| 10H1084 | China | Asia | 1 | 4 | 12 | 23 | 3 | 9 | 325 | 4 |
| 10H1087 | China | Asia | 1 | 4 | 12 | 23 | 36 | 1 | 327 | 4 |
| F2011af026 | Taiwan | Asia | 1 | 4 | 22 | 54 | 36 | 9 | 386 | 4 |
| CTEGY-01 | Egypt | Africa | 1 | 9 | 22 | 23 | 36 | 9 | 401 | 4 |
| CTEGY-24 | Egypt | Africa | 1 | 9 | 22 | 23 | 36 | 9 | 401 | 4 |
| CTEGY-28 | Egypt | Africa | 1 | 9 | 22 | 23 | 36 | 9 | 401 | 4 |
| CTEGY-52 | Egypt | Africa | 1 | 9 | 22 | 23 | 36 | 9 | 401 | 4 |
| CTEGY-56 | Egypt | Africa | 1 | 9 | 22 | 23 | 36 | 9 | 401 | 4 |
| CTEGY-57 | Egypt | Africa | 1 | 9 | 22 | 23 | 36 | 9 | 401 | 4 |
| CTEGY-62 | Egypt | Africa | 1 | 9 | 22 | 23 | 36 | 9 | 401 | 4 |
| CTEGY-67 | Egypt | Africa | 1 | 9 | 22 | 23 | 36 | 9 | 401 | 4 |
| CTEGY-68 | Egypt | Africa | 1 | 9 | 22 | 23 | 36 | 9 | 401 | 4 |
| CTEGY-71 | Egypt | Africa | 1 | 9 | 22 | 23 | 36 | 9 | 401 | 4 |
| C7852 | South Korea | Asia | 1 | 9 | 22 | 23 | 36 | 9 | 401 | 4 |
| C9280 | South Korea | Asia | 1 | 9 | 22 | 23 | 43 | 9 | 417 | 4 |
| F2013d100-3 | Taiwan | Asia | 1 | 4 | 22 | 7 | 36 | 9 | 554 | 4 |
| F2013g096 | Taiwan | Asia | 1 | 4 | 22 | 23 | 78 | 9 | 558 | 4 |
| F2014f082-1 | Taiwan | Asia | 1 | 4 | 22 | 23 | 36 | 2 | 562 | 4 |
| CT242 | Taiwan | Asia | 1 | 4 | 3 | 23 | 78 | 9 | 785 | 4 |
| C2-1030619 | Taiwan | Asia | 1 | 4 | 22 | 88 | 36 | 9 | 796 | 4 |
| F2017b014 | Taiwan | Asia | 1 | 4 | 22 | 7 | 135 | 9 | 823 | 4 |
| F2017b034 | Taiwan | Asia | 1 | 4 | 22 | 7 | 135 | 9 | 823 | 4 |
| F2017b038 | Taiwan | Asia | 1 | 9 | 22 | 23 | 135 | 9 | 825 | 4 |
| F2017d049 | Taiwan | Asia | 1 | 4 | 22 | 23 | 114 | 9 | 832 | 4 |
| F2017d081 | Taiwan | Asia | 1 | 4 | 22 | 23 | 135 | 9 | 833 | 4 |
| F2017f050 | Taiwan | Asia | 1 | 4 | 22 | 23 | 135 | 9 | 833 | 4 |
| F2017e045 | Taiwan | Asia | 1 | 4 | 22 | 23 | 135 | 2 | 834 | 4 |
| CT195 | Taiwan | Asia | 1 | 4 | 22 | 93 | 36 | 9 | 853 | 4 |
| F2015f061 | Taiwan | Asia | 1 | 4 | 22 | 7 | 135 | 1 | 870 | 4 |
| F2011af095 | Taiwan | Asia | 1 | 4 | 12 | 23 | 55 | 9 | 880 | 4 |
| T_Y48 | South Korea | Asia | 1 | 4 | 3 | 23 | 43 | 9 | 930 | 4 |
| YM180522 | Taiwan | Asia | 1 | 4 | 22 | 106 | 135 | 9 | 956 | 4 |
| F2019b030 | Taiwan | Asia | 1 | 4 | 12 | 7 | 36 | 9 | 1078 | 4 |
| F2018d051 | Taiwan | Asia | 1 | 4 | 3 | 7 | 36 | 9 | 1112 | 4 |
| F2018g063 | Taiwan | Asia | 1 | 4 | 3 | 7 | 78 | 9 | 1113 | 4 |
| F2019g051 | Taiwan | Asia | 1 | 4 | 22 | 7 | 36 | 2 | 1114 | 4 |
| F2020a052 | Taiwan | Asia | 1 | 4 | 22 | 7 | 55 | 9 | 1115 | 4 |
| F2019d052 | Taiwan | Asia | 1 | 4 | 22 | 23 | 36 | 1 | 1116 | 4 |
| F2019b013 | Taiwan | Asia | 1 | 9 | 22 | 7 | 36 | 9 | 1121 | 4 |
| CTEGY-41 | Egypt | Africa | 1 | 32 | 22 | 23 | 36 | 9 | 1169 | 4 |
| CTEGY-65 | Egypt | Africa | 1 | 32 | 22 | 23 | 36 | 9 | 1169 | 4 |
| **Abbreviations: MLST: Multi Locus Sequence Typing; DST: Diploid Sequence Type; CC: Clonal Complex.** | | | | | | | | | | |

**CC5**

| **Isolate n°** | **Country** | **Continent** | **MLST loci** | | | | | | **DST** | **CC** |
| --- | --- | --- | --- | --- | --- | --- | --- | --- | --- | --- |
|  |  |  | ***ICL1*** | ***MDR1*** | ***SAPT2*** | ***SAPT4*** | ***XYR1*** | ***ZWF1a*** |  |  |
| BZR-53 | China | Asia | 1 | 9 | 3 | 17 | 2 | 22 | 283 | 5 |
| BZR-115 | China | Asia | 1 | 9 | 12 | 17 | 2 | 2 | 316 | 5 |
| CTEGY-06 | Egypt | Africa | 1 | 22 | 12 | 17 | 60 | 22 | 331 | 5 |
| CTEGY-09 | Egypt | Africa | 1 | 22 | 12 | 17 | 60 | 22 | 331 | 5 |
| 09HX011 | China | Asia | 1 | 22 | 12 | 17 | 60 | 22 | 331 | 5 |
| 09PU031 | China | Asia | 1 | 22 | 12 | 17 | 60 | 22 | 331 | 5 |
| 10PU099 | China | Asia | 1 | 22 | 12 | 17 | 60 | 22 | 331 | 5 |
| 15_HNHK_M_76_PT | China | Asia | 1 | 22 | 12 | 17 | 60 | 22 | 331 | 5 |
| 19_HNSY_M_82_PT | China | Asia | 1 | 22 | 12 | 17 | 60 | 22 | 331 | 5 |
| 33_HNHK_W_79_PT | China | Asia | 1 | 22 | 12 | 17 | 60 | 22 | 331 | 5 |
| DFG118_hn_f_61_Pt | China | Asia | 1 | 22 | 12 | 17 | 60 | 22 | 331 | 5 |
| 10HX049 | China | Asia | 1 | 9 | 12 | 17 | 2 | 22 | 336 | 5 |
| 26_HNHK_W_79_PT | China | Asia | 1 | 9 | 12 | 17 | 2 | 22 | 336 | 5 |
| 10HX081 | China | Asia | 1 | 9 | 12 | 17 | 2 | 3 | 338 | 5 |
| 22_HNWC_M_89_PT | China | Asia | 1 | 9 | 12 | 17 | 60 | 22 | 394 | 5 |
| 3_HNHK_w_90_PT | China | Asia | 1 | 9 | 12 | 17 | 60 | 22 | 394 | 5 |
| 4_HNHK_w_65_PT | China | Asia | 1 | 9 | 12 | 17 | 60 | 22 | 394 | 5 |
| 9_HNHK_M_57_PT | China | Asia | 1 | 9 | 12 | 17 | 60 | 22 | 394 | 5 |
| C21_HNLS_M_18_HP | China | Asia | 1 | 9 | 12 | 17 | 60 | 22 | 394 | 5 |
| C23_HNLS_W_86_HP | China | Asia | 1 | 9 | 12 | 17 | 60 | 22 | 394 | 5 |
| C3_HNLS_M_19_HP | China | Asia | 1 | 9 | 12 | 17 | 60 | 22 | 394 | 5 |
| E285 | China | Asia | 1 | 9 | 12 | 17 | 60 | 22 | 394 | 5 |
| BS52 | South Korea | Asia | 1 | 9 | 12 | 17 | 60 | 22 | 394 | 5 |
| DFR36_hn_m_46_Pt | China | Asia | 1 | 9 | 3 | 14 | 60 | 22 | 427 | 5 |
| DFR35_hn_m_59_Pt | China | Asia | 1 | 9 | 3 | 14 | 60 | 22 | 427 | 5 |
| 2_HNHK_M_79_PT | China | Asia | 1 | 9 | 12 | 17 | 60 | 3 | 428 | 5 |
| 6_HNHK_M_66_PT | China | Asia | 1 | 9 | 12 | 17 | 26 | 22 | 429 | 5 |
| BT10_hn_Li_m_40_Pt | China | Asia | 1 | 9 | 12 | 14 | 60 | 22 | 430 | 5 |
| DFG11_sc_m_75_Pt | China | Asia | 1 | 9 | 12 | 14 | 60 | 22 | 430 | 5 |
| DFG14_gx_Z_m_58_Pt | China | Asia | 1 | 9 | 12 | 14 | 60 | 22 | 430 | 5 |
| HY51 | China | Asia | 1 | 9 | 12 | 14 | 60 | 22 | 430 | 5 |
| DFG56_sc_m_43_Pt | China | Asia | 1 | 9 | 12 | 62 | 60 | 22 | 432 | 5 |
| DFG55_sc_m_73_Pt | China | Asia | 1 | 9 | 12 | 62 | 60 | 22 | 432 | 5 |
| DZ56_hn_m_76_Pt | China | Asia | 1 | 9 | 12 | 17 | 85 | 22 | 433 | 5 |
| C22_HNLS_W_20_HP | China | Asia | 1 | 9 | 22 | 17 | 60 | 22 | 434 | 5 |
| 32_HNHK_M_62_PT | China | Asia | 1 | 22 | 12 | 17 | 60 | 7 | 441 | 5 |
| 5_HNHK_M_69_PT | China | Asia | 1 | 22 | 12 | 17 | 53 | 7 | 442 | 5 |
| C13_HNLS_W_80_HP | China | Asia | 1 | 22 | 12 | 17 | 60 | 31 | 443 | 5 |
| DFR52_hn_f_31_Pt | China | Asia | 1 | 22 | 12 | 14 | 60 | 7 | 444 | 5 |
| DFR52_hn_f_31_Pt | China | Asia | 1 | 22 | 12 | 14 | 60 | 7 | 444 | 5 |
| YM140912 | Taiwan | Asia | 1 | 22 | 12 | 17 | 134 | 7 | 665 | 5 |
| 8690-2 | China | Asia | 1 | 22 | 12 | 17 | 60 | 9 | 727 | 5 |
| CT268 | Taiwan | Asia | 1 | 22 | 12 | 7 | 146 | 7 | 781 | 5 |
| C2-1031211 | Taiwan | Asia | 1 | 7 | 12 | 17 | 60 | 52 | 804 | 5 |
| F2014f030 | Taiwan | Asia | 1 | 22 | 12 | 14 | 146 | 7 | 866 | 5 |
| YM140730 | Taiwan | Asia | 1 | 22 | 12 | 17 | 146 | 7 | 910 | 5 |
| 2011x059 | Taiwan | Asia | 1 | 174 | 12 | 17 | 60 | 22 | 913 | 5 |
| CTR-41 | China | Asia | 1 | 9 | 3 | 17 | 60 | 22 | 981 | 5 |
| CTR-78 | China | Asia | 1 | 7 | 12 | 17 | 60 | 31 | 986 | 5 |
| CTR-37 | China | Asia | 1 | 22 | 62 | 17 | 60 | 31 | 992 | 5 |
| F2019h044 | Taiwan | Asia | 1 | 9 | 22 | 14 | 60 | 22 | 1079 | 5 |
| F2019h049 | Taiwan | Asia | 1 | 22 | 12 | 14 | 60 | 22 | 1080 | 5 |
| F2018a090 | Taiwan | Asia | 1 | 22 | 12 | 14 | 53 | 7 | 1126 | 5 |
| **Abbreviations: MLST: Multi Locus Sequence Typing; DST: Diploid Sequence Type; CC: Clonal Complex.** | | | | | | | | | | |

**CC6**

| **Isolate n°** | **Country** | **Continent** | **MLST loci** | | | | | | **DST** | **CC** |
| --- | --- | --- | --- | --- | --- | --- | --- | --- | --- | --- |
|  |  |  | ***ICL1*** | ***MDR1*** | ***SAPT2*** | ***SAPT4*** | ***XYR1*** | ***ZWF1a*** |  |  |
| 09PU032 | China | Asia | 1 | 44 | 3 | 7 | 38 | 3 | 99 | 6 |
| AM2005/0090 | Colombia | South America | 1 | 44 | 3 | 7 | 38 | 3 | 99 | 6 |
| 30_HNHK_M_38_PT | China | Asia | 1 | 44 | 3 | 7 | 58 | 3 | 149 | 6 |
| BT20_hn_m_28_Pt | China | Asia | 1 | 44 | 3 | 7 | 58 | 3 | 149 | 6 |
| C2_HNLS_M_10_HP | China | Asia | 1 | 44 | 3 | 7 | 58 | 3 | 149 | 6 |
| C29_HNLS_W_60_HP | China | Asia | 1 | 44 | 3 | 7 | 58 | 3 | 149 | 6 |
| DFR77_hn_f_60_Pt | China | Asia | 1 | 44 | 3 | 7 | 58 | 3 | 149 | 6 |
| YM060177 | Taiwan | Asia | 1 | 44 | 3 | 7 | 58 | 3 | 149 | 6 |
| YM060097 | Taiwan | Asia | 1 | 44 | 3 | 7 | 58 | 3 | 149 | 6 |
| YM990579 | Taiwan | Asia | 1 | 44 | 3 | 7 | 58 | 3 | 149 | 6 |
| NHUE17 | Taiwan | Asia | 1 | 44 | 3 | 7 | 58 | 3 | 149 | 6 |
| NHUE18 | Taiwan | Asia | 1 | 44 | 3 | 7 | 58 | 3 | 149 | 6 |
| NHUE19 | Taiwan | Asia | 1 | 44 | 3 | 7 | 58 | 3 | 149 | 6 |
| NHUE28 | Taiwan | Asia | 1 | 44 | 3 | 7 | 58 | 3 | 149 | 6 |
| NHUE29 | Taiwan | Asia | 1 | 44 | 3 | 7 | 58 | 3 | 149 | 6 |
| NHUE30 | Taiwan | Asia | 1 | 44 | 3 | 7 | 58 | 3 | 149 | 6 |
| NHUE33 | Taiwan | Asia | 1 | 44 | 3 | 7 | 58 | 3 | 149 | 6 |
| NHUE34 | Taiwan | Asia | 1 | 44 | 3 | 7 | 58 | 3 | 149 | 6 |
| NHUE40 | Taiwan | Asia | 1 | 44 | 3 | 7 | 58 | 3 | 149 | 6 |
| 29 | Brazil | South America | 1 | 17 | 3 | 7 | 1 | 3 | 242 | 6 |
| 10HX058 | China | Asia | 1 | 44 | 1 | 7 | 38 | 3 | 337 | 6 |
| F2012f029 | Taiwan | Asia | 1 | 97 | 3 | 7 | 1 | 3 | 370 | 6 |
| CYCT2 | China | Asia | 1 | 44 | 3 | 7 | 109 | 3 | 414 | 6 |
| C18_HNLS_M_26_HP | China | Asia | 1 | 7 | 1 | 7 | 23 | 3 | 418 | 6 |
| NK191_hn_m_28_Pt | China | Asia | 1 | 7 | 1 | 7 | 38 | 3 | 419 | 6 |
| C16_HNLS_M_43_HP | China | Asia | 1 | 7 | 3 | 7 | 58 | 3 | 421 | 6 |
| C17_HNLS_W_60_HP | China | Asia | 1 | 7 | 3 | 7 | 58 | 7 | 422 | 6 |
| C38_HNLS_W_66_HP | China | Asia | 1 | 44 | 3 | 7 | 58 | 22 | 446 | 6 |
| C14_HNLS_W_13_HP | China | Asia | 38 | 44 | 3 | 7 | 58 | 3 | 501 | 6 |
| C20_HNLS_M_18_HP | China | Asia | 38 | 44 | 3 | 7 | 58 | 22 | 502 | 6 |
| CTCMR-14 | Cameroon | Africa | 1 | 44 | 1 | 7 | 58 | 3 | 522 | 6 |
| CTCMR-19 | Cameroon | Africa | 1 | 44 | 1 | 7 | 58 | 3 | 522 | 6 |
| 606 | China | Asia | 1 | 44 | 1 | 7 | 58 | 3 | 522 | 6 |
| 607 | China | Asia | 1 | 44 | 1 | 7 | 58 | 3 | 522 | 6 |
| E228 | China | Asia | 1 | 44 | 1 | 7 | 58 | 3 | 522 | 6 |
| F2013e038-1 | Taiwan | Asia | 1 | 122 | 3 | 7 | 58 | 7 | 555 | 6 |
| YFA122284 | Taiwan | Asia | 1 | 44 | 3 | 7 | 58 | 1 | 576 | 6 |
| YFA121513 | Taiwan | Asia | 1 | 44 | 3 | 7 | 124 | 3 | 577 | 6 |
| YM140797 | Taiwan | Asia | 1 | 97 | 3 | 7 | 81 | 3 | 584 | 6 |
| C2-1000502 | Taiwan | Asia | 1 | 44 | 3 | 7 | 58 | 7 | 805 | 6 |
| F2017e077 | Taiwan | Asia | 1 | 44 | 58 | 7 | 58 | 3 | 835 | 6 |
| F2014f057 | Taiwan | Asia | 1 | 44 | 3 | 7 | 154 | 3 | 867 | 6 |
| F2016f044 | Taiwan | Asia | 1 | 7 | 1 | 7 | 156 | 3 | 873 | 6 |
| YFA181245 | Taiwan | Asia | 1 | 7 | 3 | 7 | 1 | 3 | 957 | 6 |
| YM180459 | Taiwan | Asia | 1 | 44 | 4 | 7 | 58 | 3 | 960 | 6 |
| CS1477 | Singapore | Asia | 1 | 44 | 1 | 7 | 92 | 3 | 1026 | 6 |
| CW0502 | Singapore | Asia | 1 | 44 | 3 | 7 | 187 | 1 | 1027 | 6 |
| CS1861 | Singapore | Asia | 1 | 44 | 3 | 7 | 92 | 3 | 1076 | 6 |
| F2018b053 | Taiwan | Asia | 1 | 44 | 3 | 7 | 201 | 3 | 1127 | 6 |
| CTCMR-20 | Cameroon | Africa | 1 | 44 | 1 | 7 | 58 | 6 | 1182 | 6 |
| **Abbreviations: MLST: Multi Locus Sequence Typing; DST: Diploid Sequence Type; CC: Clonal Complex.** | | | | | | | | | | |

**CC7**

| **Isolate n°** | **Country** | **Continent** | **MLST loci** | | | | | | **DST** | **CC** |
| --- | --- | --- | --- | --- | --- | --- | --- | --- | --- | --- |
|  |  |  | ***ICL1*** | ***MDR1*** | ***SAPT2*** | ***SAPT4*** | ***XYR1*** | ***ZWF1a*** |  |  |
| J981379 | USA | North America | 1 | 24 | 3 | 7 | 14 | 6 | 42 | 7 |
| J981352 | USA | North America | 1 | 24 | 3 | 7 | 30 | 4 | 77 | 7 |
| AM2005/0282 | UK | Europe | 1 | 43 | 3 | 7 | 24 | 6 | 95 | 7 |
| AM2005/0564 | UK | Europe | 1 | 24 | 3 | 7 | 48 | 6 | 124 | 7 |
| 42 | Brazil | South America | 1 | 24 | 3 | 7 | 24 | 6 | 232 | 7 |
| 35 | Brazil | South America | 1 | 24 | 3 | 7 | 24 | 7 | 233 | 7 |
| 5a | Brazil | South America | 1 | 24 | 3 | 7 | 53 | 6 | 239 | 7 |
| 37 | Brazil | South America | 1 | 24 | 3 | 7 | 47 | 6 | 240 | 7 |
| 38 | Brazil | South America | 1 | 82 | 3 | 7 | 24 | 6 | 250 | 7 |
| 15 | Brazil | South America | 1 | 24 | 3 | 7 | 90 | 6 | 262 | 7 |
| YM140073 | Taiwan | Asia | 1 | 7 | 3 | 10 | 6 | 6 | 662 | 7 |
| T_Y199 | South Korea | Asia | 1 | 7 | 3 | 7 | 14 | 6 | 932 | 7 |
| T_Y190 | South Korea | Asia | 1 | 7 | 3 | 7 | 24 | 6 | 933 | 7 |
| T_32538 | South Korea | Asia | 1 | 24 | 3 | 7 | 48 | 7 | 936 | 7 |
| T_Y399 | South Korea | Asia | 1 | 24 | 3 | 7 | 125 | 6 | 937 | 7 |
| SP4433 | Canada | North America | 1 | 24 | 3 | 7 | 85 | 6 | 974 | 7 |
| SP4501 | Canada | North America | 1 | 24 | 3 | 7 | 85 | 6 | 974 | 7 |
| CTEGY-32 | Egypt | Africa | 1 | 7 | 3 | 7 | 76 | 6 | 1167 | 7 |
| CTEGY-63 | Egypt | Africa | 1 | 7 | 3 | 10 | 76 | 6 | 1173 | 7 |
| CTCMR-05 | Cameroon | Africa | 1 | 7 | 3 | 10 | 73 | 6 | 1175 | 7 |
| CTEGY-74 | Egypt | Africa | 1 | 7 | 3 | 10 | 73 | 6 | 1175 | 7 |
| CTCMR-02 | Cameroon | Africa | 1 | 7 | 3 | 7 | 73 | 6 | 1176 | 7 |
| IFM61175 | Japan | Asia | 1 | 202 | 3 | 7 | 24 | 6 | 1204 | 7 |
| IFM61749 | Japan | Asia | 1 | 24 | 3 | 7 | 24 | 4 | 1208 | 7 |
| IFM63625 | Japan | Asia | 1 | 203 | 3 | 7 | 24 | 4 | 1210 | 7 |
| IFM65231 | Japan | Asia | 1 | 24 | 3 | 7 | 24 | 69 | 1212 | 7 |
| **Abbreviations: MLST: Multi Locus Sequence Typing; DST: Diploid Sequence Type; CC: Clonal Complex.** | | | | | | | | | | |

**CC9**

| **Isolate n°** | **Country** | **Continent** | **MLST loci** | | | | | | **DST** | **CC** |
| --- | --- | --- | --- | --- | --- | --- | --- | --- | --- | --- |
|  |  |  | ***ICL1*** | ***MDR1*** | ***SAPT2*** | ***SAPT4*** | ***XYR1*** | ***ZWF1a*** |  |  |
| J941839 | The Netherlands | Europe | 1 | 17 | 2 | 14 | 1 | 7 | 26 | 9 |
| AM2005/0092 | Colombia | South America | 1 | 17 | 2 | 14 | 23 | 3 | 100 | 9 |
| AM2005/0095 | Colombia | South America | 1 | 17 | 2 | 14 | 23 | 3 | 100 | 9 |
| YM020671 | Taiwan | Asia | 1 | 17 | 2 | 14 | 63 | 3 | 158 | 9 |
| 10TZ071 | China | Asia | 1 | 17 | 2 | 14 | 1 | 3 | 344 | 9 |
| F2012h009 | Taiwan | Asia | 1 | 17 | 2 | 14 | 100 | 3 | 359 | 9 |
| IRCCS 46 | Italy | Europe | 1 | 17 | 2 | 14 | 100 | 3 | 359 | 9 |
| IRCCS 47 | Italy | Europe | 1 | 17 | 2 | 14 | 100 | 3 | 359 | 9 |
| CTRC-02 | Italy | Europe | 1 | 17 | 2 | 14 | 100 | 3 | 359 | 9 |
| CTRC-03 | Italy | Europe | 1 | 17 | 2 | 14 | 100 | 3 | 359 | 9 |
| C10056 | South Korea | Asia | 1 | 19 | 2 | 14 | 100 | 3 | 404 | 9 |
| DZ49_hn_m_85_Pt | China | Asia | 1 | 7 | 2 | 14 | 100 | 3 | 420 | 9 |
| 9483 | China | Asia | 1 | 7 | 2 | 14 | 100 | 3 | 420 | 9 |
| 608 | China | Asia | 1 | 22 | 2 | 14 | 53 | 3 | 523 | 9 |
| 609 | China | Asia | 1 | 17 | 2 | 14 | 60 | 43 | 524 | 9 |
| F2011v063-1 | Taiwan | Asia | 1 | 124 | 2 | 14 | 60 | 3 | 549 | 9 |
| F2011v063-2 | Taiwan | Asia | 1 | 17 | 2 | 14 | 100 | 7 | 550 | 9 |
| IRCCS 14 | Italy | Europe | 1 | 17 | 2 | 14 | 53 | 3 | 750 | 9 |
| C2-1011206 | Taiwan | Asia | 1 | 7 | 2 | 14 | 100 | 7 | 808 | 9 |
| C2-1020810 | Taiwan | Asia | 1 | 17 | 4 | 14 | 100 | 3 | 809 | 9 |
| C2-1030813 | Taiwan | Asia | 1 | 17 | 4 | 14 | 100 | 3 | 809 | 9 |
| C2-1021101 | Taiwan | Asia | 1 | 17 | 2 | 14 | 60 | 3 | 811 | 9 |
| YM140438 | Taiwan | Asia | 1 | 17 | 22 | 14 | 100 | 3 | 923 | 9 |
| CS2397 | Singapore | Asia | 1 | 17 | 2 | 14 | 190 | 3 | 1020 | 9 |
| **Abbreviations: MLST: Multi Locus Sequence Typing; DST: Diploid Sequence Type; CC: Clonal Complex.** | | | | | | | | | | |

**CC15**

| **Isolate n°** | **Country** | **Continent** | **MLST loci** | | | | | | **DST** | **CC** |
| --- | --- | --- | --- | --- | --- | --- | --- | --- | --- | --- |
|  |  |  | ***ICL1*** | ***MDR1*** | ***SAPT2*** | ***SAPT4*** | ***XYR1*** | ***ZWF1a*** |  |  |
| CTCMR-10 | Cameroon | Africa | 3 | 4 | 1 | 41 | 77 | 4 | 346 | 15 |
| CTEGY-18 | Egypt | Africa | 3 | 4 | 3 | 41 | 77 | 4 | 985 | 15 |
| BZR-98 | China | Asia | 10 | 4 | 4 | 41 | 77 | 4 | 310 | 15 |
| BZR-117 | China | Asia | 3 | 4 | 4 | 41 | 77 | 4 | 318 | 15 |
| 09TZ034 | China | Asia | 3 | 4 | 1 | 41 | 77 | 7 | 341 | 15 |
| 09PU016 | China | Asia | 3 | 4 | 1 | 41 | 77 | 4 | 346 | 15 |
| C30_HNLS_M_10_HP | China | Asia | 3 | 4 | 1 | 41 | 77 | 4 | 346 | 15 |
| DZ11_hn_m_2_Pt | China | Asia | 3 | 4 | 1 | 41 | 77 | 4 | 346 | 15 |
| DZ50_hn_m_94_Pt | China | Asia | 3 | 4 | 1 | 41 | 77 | 4 | 346 | 15 |
| 8630-2 | China | Asia | 3 | 4 | 1 | 41 | 77 | 4 | 346 | 15 |
| 569 | China | Asia | 10 | 4 | 1 | 41 | 117 | 4 | 503 | 15 |
| 570 | China | Asia | 10 | 4 | 1 | 41 | 77 | 4 | 504 | 15 |
| 571 | China | Asia | 10 | 4 | 1 | 41 | 77 | 4 | 504 | 15 |
| 600 | China | Asia | 3 | 32 | 1 | 41 | 77 | 4 | 519 | 15 |
| CTR-72 | China | Asia | 3 | 4 | 3 | 41 | 77 | 4 | 985 | 15 |
| 4a | Brazil | South America | 3 | 4 | 4 | 17 | 77 | 4 | 264 | 15 |
| **Abbreviations: MLST: Multi Locus Sequence Typing; DST: Diploid Sequence Type; CC: Clonal Complex.** | | | | | | | | | | |

**CC29**

| **Isolate n°** | **Country** | **Continent** | **MLST loci** | | | | | | **DST** | **CC** |
| --- | --- | --- | --- | --- | --- | --- | --- | --- | --- | --- |
|  |  |  | ***ICL1*** | ***MDR1*** | ***SAPT2*** | ***SAPT4*** | ***XYR1*** | ***ZWF1a*** |  |  |
| BT24_hn_Li_f_16_Pt | China | Asia | 3 | 22 | 1 | 10 | 27 | 1 | 487 | 29 |
| DZ27_hn_m_3_Pt | China | Asia | 3 | 22 | 1 | 3 | 9 | 1 | 488 | 29 |
| DZ51_hn_m_92_Pt | China | Asia | 3 | 22 | 1 | 3 | 27 | 1 | 489 | 29 |
| E610 | China | Asia | 3 | 22 | 1 | 3 | 27 | 1 | 489 | 29 |
| E610-2 | China | Asia | 3 | 22 | 1 | 3 | 27 | 1 | 489 | 29 |
| DFZ111_hn_f_55_Pt | China | Asia | 3 | 114 | 1 | 3 | 27 | 1 | 495 | 29 |
| CTR-85 | China | Asia | 3 | 22 | 1 | 3 | 27 | 65 | 1002 | 29 |
| CTEGY-14 | Egypt | Africa | 3 | 22 | 68 | 3 | 27 | 1 | 1162 | 29 |
| **Abbreviations: MLST: Multi Locus Sequence Typing; DST: Diploid Sequence Type; CC: Clonal Complex.** | | | | | | | | | | |

**CC30**

| **Isolate n°** | **Country** | **Continent** | **MLST loci** | | | | | | **DST** | **CC** |
| --- | --- | --- | --- | --- | --- | --- | --- | --- | --- | --- |
|  |  |  | ***ICL1*** | ***MDR1*** | ***SAPT2*** | ***SAPT4*** | ***XYR1*** | ***ZWF1a*** |  |  |
| CTEGY-22 | Egypt | Africa | 1 | 59 | 1 | 3 | 3 | 1 | 1164 | 30 |
| 09TZ029 | China | Asia | 20 | 59 | 1 | 10 | 3 | 1 | 164 | 30 |
| DOH15 | Taiwan | Asia | 20 | 59 | 1 | 10 | 3 | 1 | 164 | 30 |
| DOH44 | Taiwan | Asia | 20 | 59 | 1 | 10 | 3 | 1 | 164 | 30 |
| DOH58 | Taiwan | Asia | 20 | 59 | 1 | 10 | 3 | 1 | 164 | 30 |
| DOH64 | Taiwan | Asia | 20 | 59 | 1 | 10 | 3 | 1 | 164 | 30 |
| DOH66 | Taiwan | Asia | 20 | 59 | 1 | 10 | 3 | 1 | 164 | 30 |
| DOH74 | Taiwan | Asia | 20 | 59 | 1 | 10 | 3 | 1 | 164 | 30 |
| DOH77 | Taiwan | Asia | 20 | 59 | 1 | 10 | 3 | 1 | 164 | 30 |
| DOH94 | Taiwan | Asia | 20 | 59 | 1 | 10 | 3 | 1 | 164 | 30 |
| DOH54 | Taiwan | Asia | 20 | 59 | 1 | 10 | 3 | 1 | 164 | 30 |
| DOH65 | Taiwan | Asia | 20 | 59 | 1 | 10 | 3 | 1 | 164 | 30 |
| YM060141 | Taiwan | Asia | 1 | 59 | 1 | 10 | 3 | 1 | 192 | 30 |
| F2014g077 | Taiwan | Asia | 1 | 69 | 1 | 7 | 3 | 1 | 883 | 30 |
| F2011r070 | Taiwan | Asia | 1 | 59 | 1 | 7 | 3 | 1 | 1081 | 30 |
| SCS74663 | UK | Europe | 1 | 52 | 1 | 3 | 3 | 1 | 130 | 30 |
| **Abbreviations: MLST: Multi Locus Sequence Typing; DST: Diploid Sequence Type; CC: Clonal Complex.** | | | | | | | | | | |

**CC36**

| **Isolate n°** | **Country** | **Continent** | **MLST loci** | | | | | | **DST** | **CC** |
| --- | --- | --- | --- | --- | --- | --- | --- | --- | --- | --- |
|  |  |  | ***ICL1*** | ***MDR1*** | ***SAPT2*** | ***SAPT4*** | ***XYR1*** | ***ZWF1a*** |  |  |
| IRCCS 1 | Italy | Europe | 17 | 93 | 29 | 83 | 138 | 30 | 747 | 36 |
| IRCCS 2 | Italy | Europe | 17 | 93 | 29 | 83 | 138 | 30 | 747 | 36 |
| IRCCS 15 | Italy | Europe | 17 | 93 | 29 | 83 | 138 | 30 | 747 | 36 |
| IRCCS 23 | Italy | Europe | 17 | 93 | 29 | 83 | 138 | 30 | 747 | 36 |
| IRCCS 24 | Italy | Europe | 17 | 93 | 29 | 83 | 138 | 30 | 747 | 36 |
| IRCCS 31 | Italy | Europe | 17 | 93 | 29 | 83 | 138 | 30 | 747 | 36 |
| IRCCS 32 | Italy | Europe | 17 | 93 | 29 | 83 | 138 | 30 | 747 | 36 |
| IRCCS 37 | Italy | Europe | 17 | 93 | 29 | 83 | 138 | 30 | 747 | 36 |
| IRCCS 39 | Italy | Europe | 17 | 93 | 29 | 83 | 138 | 30 | 747 | 36 |
| C1T1V | Italy | Europe | 17 | 93 | 29 | 83 | 138 | 30 | 747 | 36 |
| OSS1C-R | Italy | Europe | 17 | 93 | 29 | 83 | 138 | 30 | 747 | 36 |
| IRXLP2 | Italy | Europe | 17 | 93 | 29 | 83 | 138 | 30 | 747 | 36 |
| IRCCS 7 | Italy | Europe | 17 | 93 | 29 | 82 | 138 | 30 | 748 | 36 |
| IRCCS 8 | Italy | Europe | 17 | 93 | 29 | 82 | 138 | 30 | 748 | 36 |
| IRCCS 38 | Italy | Europe | 17 | 93 | 53 | 83 | 138 | 30 | 749 | 36 |
| IRCCS 51 | Italy | Europe | 17 | 93 | 29 | 83 | 2 | 30 | 759 | 36 |
| IRCCS 52 | Italy | Europe | 17 | 93 | 29 | 83 | 2 | 30 | 759 | 36 |
| CTRC-04 | Italy | Europe | 47 | 93 | 29 | 82 | 138 | 30 | 917 | 36 |
| **Abbreviations: MLST: Multi Locus Sequence Typing; DST: Diploid Sequence Type; CC: Clonal Complex.** | | | | | | | | | | |

**CC62**

| **Isolate n°** | **Country** | **Continent** | **MLST loci** | | | | | | **DST** | **CC** |
| --- | --- | --- | --- | --- | --- | --- | --- | --- | --- | --- |
|  |  |  | ***ICL1*** | ***MDR1*** | ***SAPT2*** | ***SAPT4*** | ***XYR1*** | ***ZWF1a*** |  |  |
| CTEGY-07 | Egypt | Africa | 1 | 42 | 1 | 7 | 54 | 1 | 1165 | 62 |
| CTEGY-08 | Egypt | Africa | 1 | 42 | 1 | 7 | 54 | 1 | 1165 | 62 |
| CTEGY-25 | Egypt | Africa | 1 | 42 | 1 | 7 | 54 | 1 | 1165 | 62 |
| CTEGY-29 | Egypt | Africa | 1 | 42 | 1 | 7 | 54 | 1 | 1165 | 62 |
| CTEGY-40 | Egypt | Africa | 1 | 42 | 1 | 7 | 54 | 1 | 1165 | 62 |
| AM2005/0543 | UK | Europe | 1 | 42 | 1 | 23 | 9 | 1 | 94 | 62 |
| AM2005/0271 | UK | Europe | 1 | 42 | 1 | 23 | 9 | 1 | 94 | 62 |
| CTEGY 31 | Egypt | Africa | 1 | 42 | 1 | 23 | 54 | 1 | 689 | 62 |
| **Abbreviations: MLST: Multi Locus Sequence Typing; DST: Diploid Sequence Type; CC: Clonal Complex.** | | | | | | | | | | |

**CC63**

| **Isolate n°** | **Country** | **Continent** | **MLST loci** | | | | | | **DST** | **CC** |
| --- | --- | --- | --- | --- | --- | --- | --- | --- | --- | --- |
|  |  |  | ***ICL1*** | ***MDR1*** | ***SAPT2*** | ***SAPT4*** | ***XYR1*** | ***ZWF1a*** |  |  |
| CTCMR-03 | Cameroon | Africa | 3 | 148 | 12 | 11 | 74 | 3 | 1186 | 63 |
| CTCMR-04 | Cameroon | Africa | 3 | 148 | 12 | 11 | 74 | 3 | 1186 | 63 |
| CTCMR-16 | Cameroon | Africa | 3 | 198 | 12 | 11 | 74 | 3 | 1196 | 63 |
| G315 | China | Asia | 3 | 148 | 12 | 11 | 74 | 1 | 731 | 63 |
| **Abbreviations: MLST: Multi Locus Sequence Typing; DST: Diploid Sequence Type; CC: Clonal Complex.** | | | | | | | | | | |

**CC64**

| **Isolate n°** | **Country** | **Continent** | **MLST loci** | | | | | | **DST** | **CC** |
| --- | --- | --- | --- | --- | --- | --- | --- | --- | --- | --- |
|  |  |  | ***ICL1*** | ***MDR1*** | ***SAPT2*** | ***SAPT4*** | ***XYR1*** | ***ZWF1a*** |  |  |
| CTCMR-13 | Cameroon | Africa | 1 | 39 | 3 | 7 | 80 | 17 | 1179 | 64 |
| CTCMR-15 | Cameroon | Africa | 1 | 39 | 3 | 7 | 80 | 17 | 1179 | 64 |
| CTCMR-46 | Cameroon | Africa | 1 | 39 | 3 | 7 | 80 | 17 | 1179 | 64 |
| CTCMR-50 | Cameroon | Africa | 1 | 39 | 3 | 7 | 80 | 17 | 1179 | 64 |
| CTCMR-51 | Cameroon | Africa | 1 | 39 | 3 | 7 | 80 | 17 | 1179 | 64 |
| CTCMR-58 | Cameroon | Africa | 1 | 39 | 3 | 7 | 80 | 17 | 1179 | 64 |
| CTCMR-40 | Cameroon | Africa | 1 | 39 | 3 | 7 | 80 | 9 | 1185 | 64 |
| AM2005/0004 | UK | Europe | 1 | 39 | 3 | 7 | 31 | 17 | 83 | 64 |
| AM2005/0545 | UK | Europe | 1 | 39 | 3 | 7 | 31 | 17 | 83 | 64 |
| AM2005/0258 | UK | Europe | 1 | 39 | 3 | 7 | 31 | 17 | 83 | 64 |
| **Abbreviations: MLST: Multi Locus Sequence Typing; DST: Diploid Sequence Type; CC: Clonal Complex.** | | | | | | | | | | |

**CC106**

| **Isolate n°** | **Country** | **Continent** | **MLST loci** | | | | | | **DST** | **CC** |
| --- | --- | --- | --- | --- | --- | --- | --- | --- | --- | --- |
|  |  |  | ***ICL1*** | ***MDR1*** | ***SAPT2*** | ***SAPT4*** | ***XYR1*** | ***ZWF1a*** |  |  |
| CTR-56 | China | Asia | 3 | 9 | 3 | 8 | 169 | 6 | 995 | 106 |
| CBS2317 | Russia | Asia | 3 | 9 | 3 | 8 | 60 | 6 | 904 | 106 |
| **Abbreviations: MLST: Multi Locus Sequence Typing; DST: Diploid Sequence Type; CC: Clonal Complex.** | | | | | | | | | | |

**CC107**

| **Isolate n°** | **Country** | **Continent** | **MLST loci** | | | | | | **DST** | **CC** |
| --- | --- | --- | --- | --- | --- | --- | --- | --- | --- | --- |
|  |  |  | ***ICL1*** | ***MDR1*** | ***SAPT2*** | ***SAPT4*** | ***XYR1*** | ***ZWF1a*** |  |  |
| AM2005/0558 | UK | Europe | 1 | 49 | 10 | 1 | 44 | 1 | 118 | 107 |
| CTRC-01 | Italy | Europe | 1 | 49 | 10 | 1 | 161 | 1 | 915 | 107 |
| **Abbreviations: MLST: Multi Locus Sequence Typing; DST: Diploid Sequence Type; CC: Clonal Complex.** | | | | | | | | | | |

**CC108**

| **Isolate n°** | **Country** | **Continent** | **MLST loci** | | | | | | **DST** | **CC** |
| --- | --- | --- | --- | --- | --- | --- | --- | --- | --- | --- |
|  |  |  | ***ICL1*** | ***MDR1*** | ***SAPT2*** | ***SAPT4*** | ***XYR1*** | ***ZWF1a*** |  |  |
| CTRC-07 | Italy | Europe | 3 | 3 | 12 | 10 | 16 | 3 | 916 | 108 |
| CTRC-09 | Italy | Europe | 3 | 3 | 12 | 10 | 54 | 3 | 919 | 108 |
| **Abbreviations: MLST: Multi Locus Sequence Typing; DST: Diploid Sequence Type; CC: Clonal Complex.** | | | | | | | | | | |

**CC109**

| **Isolate n°** | **Country** | **Continent** | **MLST loci** | | | | | | **DST** | **CC** |
| --- | --- | --- | --- | --- | --- | --- | --- | --- | --- | --- |
|  |  |  | ***ICL1*** | ***MDR1*** | ***SAPT2*** | ***SAPT4*** | ***XYR1*** | ***ZWF1a*** |  |  |
| C10054 | South Korea | Asia | 15 | 91 | 29 | 7 | 105 | 38 | 403 | 109 |
| CTRC-10 | Italy | Europe | 15 | 91 | 29 | 102 | 105 | 38 | 920 | 109 |
| **Abbreviations: MLST: Multi Locus Sequence Typing; DST: Diploid Sequence Type; CC: Clonal Complex.** | | | | | | | | | | |
