# Supplementary material for: Multilocus sequence typing (MLST) analysis reveals many novel genotypes and a high level of genetic diversity in Candida tropicalis isolates from Italy and Africa
Source: Mycoses. 2022 Jul 7;65(11):989–1000. doi: 10.1111/myc.13483 (PMC9796097; doi:10.1111/myc.13483)
Supplement: Supplementary file 3 — Table S3 [file MYC-65-989-s001.docx]

| **Supplementary Table S3. List of DSTs statistically associated (p-value ≤0.05) with non-susceptible isolates recovered in this study, including those for which at least a MIC value was submitted in the MLST database. Yellow: Resistant isolates; Gray: SDD isolates; Green: Intermediate isolates; Blue: NWT isolates. Bold DSTs were from this study.** | | | | | | | |
| --- | --- | --- | --- | --- | --- | --- | --- |
| **Anidulafungin** | **Caspofungin** | **Fluconazole** | **Flucytosine** | **Itraconazole** | **Micafungin** | **Posaconazole** | **Voriconazole** |
| DST834 | DST3 | DST13 | DST8 | DST220 | DST75 | **DST1179** | **DST401** |
| DST1141 | **DST522** | DST606 | DST12 | DST507 | DST852 |  | **DST1175** |
| **DST1179** | **DST1186** | DST98 | DST13 | DST508 | **DST1176** |  | **DST1177** |
| **DST1184** | DST65 | DST149 | DST15 |  | **DST1184** |  | **DST1186** |
| DST852 | DST75 | DST507 | DST14 |  | DST834 |  | DST499 |
| DST974 | DST83 | **DST140** | DST17 |  | DST974 |  | DST506 |
|  | DST834 |  | DST26 |  | **DST1179** |  | DST507 |
|  | DST852 |  | DST28 |  |  |  |  |
|  | **DST1176** |  | DST56 |  |  |  |  |
|  | **DST1184** |  | DST67 |  |  |  |  |
|  |  |  | DST69 |  |  |  |  |
|  |  |  | **DST1186** |  |  |  |  |
